# Automated lesion segmentation with BIANCA: Impact of population-level features, classification algorithm and locally adaptive thresholding

**DOI:** 10.1016/j.neuroimage.2019.116056

**Published:** 2019-11-15

**Authors:** Vaanathi Sundaresan, Giovanna Zamboni, Campbell Le Heron, Peter M. Rothwell, Masud Husain, Marco Battaglini, Nicola De Stefano, Mark Jenkinson, Ludovica Griffanti

**Affiliations:** a Wellcome Centre for Integrative Neuroimaging, Oxford Centre for Functional MRI of the Brain, Nuffield Department of Clinical Neurosciences, University of Oxford, UK; b Oxford-Nottingham Centre for Doctoral Training in Biomedical Imaging, University of Oxford, UK; c Oxford India Centre for Sustainable Development, Somerville College, University of Oxford, UK; d Centre for Prevention of Stroke and Dementia, Nuffield Department of Clinical Neurosciences, University of Oxford, UK; e Nuffield Department of Clinical Neurosciences, University of Oxford, Oxford, UK; f Department of Experimental Psychology, University of Oxford, Oxford, UK; g New Zealand Brain Research Institute, Christchurch 8011, New Zealand; h Wellcome Centre for Integrative NeuroImaging, University of Oxford, UK; i Department of Medicine, Surgery and Neuroscience, University of Siena, Siena, Italy

**Keywords:** White matter hyperintensities, Structural MRI, Lesion probability map, Thresholding, Machine learning, Lesion segmentation

## Abstract

White matter hyperintensities (WMH) or white matter lesions exhibit high variability in their characteristics both at population- and subject-level, making their detection a challenging task. Population-level factors such as age, vascular risk factors and neurodegenerative diseases affect lesion load and spatial distribution. At the individual level, WMH vary in contrast, amount and distribution in different white matter regions.

In this work, we aimed to improve BIANCA, the FSL tool for WMH segmentation, in order to better deal with these sources of variability. We worked on two stages of BIANCA by improving the lesion probability map estimation (classification stage) and making the lesion probability map thresholding stage automated and adaptive to local lesion probabilities. Firstly, in order to take into account the effect of population-level factors, we included population-level lesion probabilities, modelled with respect to a parametric factor (e.g. age), in the classification stage. Secondly, we tested BIANCA performance when using four alternative classifiers commonly used in the literature with respect to K-nearest neighbour algorithm (currently used for lesion probability map estimation in BIANCA). Finally, we propose LOCally Adaptive Threshold Estimation (LOCATE), a supervised method for determining optimal local thresholds to apply to the estimated lesion probability map, as an alternative option to global thresholding (i.e. applying the same threshold to the entire lesion probability map). For these experiments we used data from a neurodegenerative cohort, a vascular cohort and the cohorts available publicly as a part of a segmentation challenge.

We observed that including population-level parametric lesion probabilities with respect to age and using alternative machine learning techniques provided negligible improvement. However, LOCATE provided a substantial improvement in the lesion segmentation performance, when compared to the global thresholding. It allowed to detect more deep lesions and provided better segmentation of periventricular lesion boundaries, despite the differences in the lesion spatial distribution and load across datasets. We further validated LOCATE on a cohort of CADASIL (Cerebral autosomal dominant arteriopathy with subcortical infarcts and leukoencephalopathy) patients, a genetic form of cerebral small vessel disease, and healthy controls, showing that LOCATE adapts well to wide variations in lesion load and spatial distribution.

## Highlights

• We aim to improve BIANCA to handle population- and subject-wise lesion variability.
• Age-based population lesion probability maps did not improve BIANCA performance.
• Among 5 classifiers tested, K-Nearest Neighbour still gives the best performance.
• LOCally Adaptive Threshold Estimation (LOCATE) proposed for lesion probability maps.
• LOCATE improves lesion segmentation and adapts well to lesion load and distribution.

## 1 Introduction

White matter hyperintensities of presumed vascular origin (WMH, also known as white matter lesions Wardlaw et al., 2013) are common radiological abnormalities often associated with cognitive impairment and one of the main signs of cerebral small vessel disease (SVD) (Pantoni, 2010). However, despite their assumed clinical importance based on their spatial location (Duchesnay et al., 2018; De Guio et al., 2018; Kim et al., 2008), accurate automated detection of WMH is very challenging due to the high variability of their characteristics both between- and within-subjects. For example, at the population-level, the amount and distribution of white matter lesions have been associated with various factors such as cognition, vascular risk factors and neurodegenerative diseases (Li et al., 2013; Debette and Markus, 2010). WMH also occur more commonly at older age (Simoni et al., 2012; Vannorsdall et al., 2009). At the subject-level, generally WMH exhibit spatial heterogeneity and do not appear with the same contrast in all regions of the brain on structural MRI images (Hernández et al., 2017). For example, typically, periventricular WMH are brighter on T2-weighted, FLAIR or proton density images and bigger compared to deep ones (Kim et al., 2008), and hence can be detected more easily.

The variability in lesion characteristics can affect the performance of supervised lesion segmentation methods at different stages. The estimation of the probability for each voxel to be a lesion (lesion probability map) is affected by population-level factors (such as age, cognition, vascular risk factors and pathological conditions) (Rostrup et al., 2012; Zamboni et al., 2017) and lesion load (Dyrby et al., 2008; Anbeek et al., 2004). Once a lesion probability map is generated, the choice of the threshold used to obtain binary maps is affected by lesion load and the variation in local intensities. In fact, heterogeneity in signal intensity affects lesion probability values determined by segmentation algorithms (De Boer et al., 2009; Anbeek et al., 2004; Dyrby et al., 2008), since brighter lesions (e.g. periventricular WMH) are typically assigned higher probabilities than those with lower contrast (e.g. deep WMH). This makes it difficult to determine the optimal threshold for obtaining the final binary lesion maps. In fact, applying a global threshold (i.e. the same threshold to all the voxels of the lesion probability map) typically results either in the exclusion of deep lesions or overestimation of periventricular lesions. This often results in a trade-off between sensitivity and specificity of lesion detection (Anbeek et al., 2004). An alternative proposed by Ling et al. (2018) consists of determining thresholds based on Fazekas score or by visual inspection of individual subjects. However, these thresholds would also be global and determining them would require manual intervention.

In this work, our objective is to improve our recently developed WMH segmentation method, Brain Intensity AbNormality Classification Algorithm (BIANCA, Griffanti et al., 2016) to overcome population- and subject-level variability in the lesion characteristics. We aim to achieve this by systematically exploring ways of improving various stages of the lesion probability map estimation, and also by developing a method for thresholding that takes into account spatial variability of lesion probability map.

Regarding the estimation of lesion probability map, we tested the inclusion of population-level lesion probabilities with respect to a parametric factor (in our case age) and explored the performance of alternative machine learning methods.

One way to include population-level probabilities is to obtain the distribution of lesions within a population with respect to a parametric factor and use it in BIANCA as either a spatial prior or an additional feature in the WMH segmentation. In this work, we modelled the spatial distribution of lesions within a population with respect to age to obtain a population-level parametric lesion probability map (PPLPM) as described in our previous study (Sundaresan et al., 2019) and explored whether the information provided by the PPLPM could improve the results or, conversely, introduce a bias towards the expected distribution (refer to Section *Use of population-level parametric lesion probability map(PPLPM)* and corresponding results).

Regarding the alternative machine learning techniques, we explored four other supervised classifiers used in the existing literature and compared their segmentation results with those obtained from the existing K-nearest neighbour (KNN) classification algorithm in BIANCA (refer to Section *Comparison of alternative classifiers within BIANCA* and results). Neural networks (Dyrby et al., 2008), support vector machines and adaboost classifier (Wen and Sachdev, 2004) have been already used for WMH segmentation, while random forests have been used for the segmentation of various structures and pathological signs that have similar characteristics as WMH on structural MR images (Geremia et al., 2011; Mitra et al., 2014; Yamamoto et al., 2010).

Aiming to improve the thresholding stage, we propose LOCATE (LOCally Adaptive Thresholds Estimation), a supervised method to determine optimal local thresholds for binarising the subject-level lesion probability map to take into account the spatial heterogeneity in lesion probabilities. LOCATE is guided by local probability values and exploits different lesion characteristics to determine local thresholds. In this work we present LOCATE with specific applicability to BIANCA, however, in principle LOCATE can be applied to the lesion probability map obtained by any method, provided the availability of a training data with manual lesion masks. We evaluated LOCATE on various clinical datasets: a neurodegenerative cohort and a vascular cohort, and a publicly available WMH segmentation challenge dataset, to observe the performance of LOCATE on data acquired under different protocols, and later applied it on two additional datasets to further explore the potential of LOCATE to provide relevant local thresholds for different lesion loads, distribution and patterns (refer to Section *LOCally Adaptive Threshold Estimation (LOCATE)* and corresponding results).

**Fig. 1:**
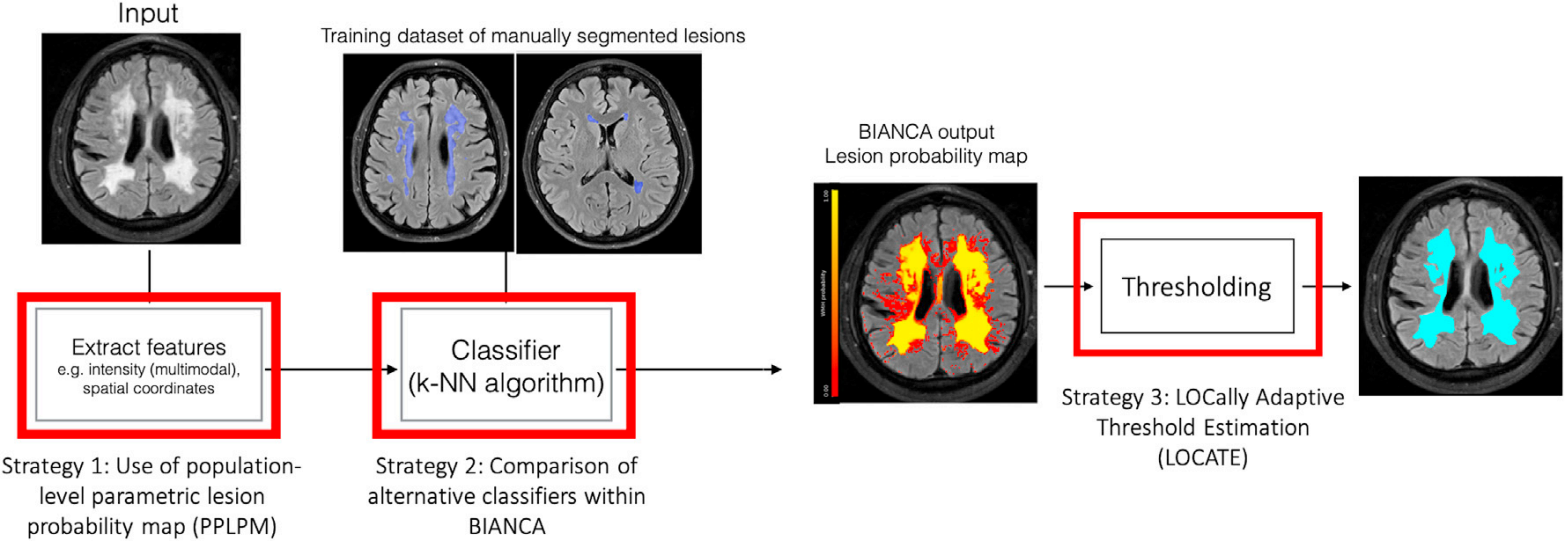
Block diagram showing the three strategies explored for improving BIANCA performance. The red highlighted blocks indicate the steps we focused on in this work.

Fig. 1 shows the block diagram describing the strategies explored for improving BIANCA performance.

## 2 Materials and methods

### 2.1 Datasets

In this work we used five datasets to study the effect of variations in lesion characteristics on the WMH segmentation. The datasets are diverse in terms of population, and therefore WMH load and characteristics.

#### 2.1.1 Dataset 1: neurodegenerative cohort (NDGEN)

This is a subset of the same dataset we used in our previous work (Griffanti et al., 2016) to optimise BIANCA (21 subjects with manual lesion segmentation available - lesion load range: 1878–89259 mm^3^, median: 20772  mm^3^). Briefly, the dataset includes MRI data from 9 subjects with probable Alzheimer’s Disease, 5 with amnestic mild cognitive impairment and 7 cognitively healthy control subjects recruited from the OPTIMA study and from the Memory Assessment Clinic at the John Radcliffe Hospital in Oxford (Zamboni et al., 2013) (age range 63–86 years, mean age 77.1 ± 5.8 years, median age 77 years, F:M = 10:11, brain volume range: 1189282–1614799 mm^3^, median: 1424669 mm^3^). MRI images were acquired at the University of Oxford OCMR centre on a 3 T S Trio scanner using a T2-weighted, fluid-attenuated inversion recovery (FLAIR) research sequence (TR/TE = 9000/89 ms, flip angle 150°, FOV 220 mm, matrix size 256×256×35, voxel size 1.1×0.9×3 mm) and a high-resolution T1-weighted images (3D MP-RAGE) were also acquired (TR/TE = 2040/4.7 ms, flip angle $8^{o}$, FOV 192 mm, matrix size 174×192×192, voxel size 1 mm isotropic) (see Griffanti et al., 2016 for more details).

#### 2.1.2 Dataset 2: vascular cohort - Oxford vascular study (OXVASC)

The dataset consists of 18 consecutive eligible participants in the OXVASC study (Rothwell et al., 2004), who had recently experienced a minor non-disabling stroke or transient ischemic attack (age range 50–91 years, mean age 73.27 ± 12.32 years, median age 75.5 years, F:M = 7:11, brain volume range: 1290926–1918604  mm^3^, median: 1568233  mm^3^). Scanning was performed at the Oxford Acute Vascular Imaging Centre (AVIC) on a 3 T S Verio scanner using a T2-weighted, FLAIR sequence (TR/TE = 9000/88 ms, flip angle 150°, FOV 192 mm, matrix size 174×52×192, voxel size 1×3×1 mm) and a high resolution T1-weighted sequence (3D MP-RAGE sequence, TR/TE = 2000/1.94 ms, flip angle $8^{o}$, FOV 256 mm, matrix size 208×256×256, voxel size 1 mm isotropic), and diffusion-weighted imaging (TR/TE = 8000/86 ms, GRAPPA factor 2, flip angle $16^{o}$, FOV 192 mm, voxel-size 2×2×2 mm, 32 directions, b-value 1500 s/mm^2^) and diffusion-weighted imaging (TR/TE = 8000/86 ms, GRAPPA factor 2, flip angle $16^{o}$, FOV 192 mm, voxel size 2×2×2 mm, 32 directions, b value 1500 s/mm^2^). Manual lesion segmentation was available for all 18 images (lesion load range: 3530–83391  mm^3^, median: 16906  mm^3^).

#### 2.1.3 Dataset 3: cerebral autosomal dominant arteriopathy with subcortical infarcts and leukoencephalopathy (CADASIL) cohort

The dataset consists of 15 patients with CADASIL (age range 33–70 years, mean age 53.73 ± 11.31 years, median age 55.5 years, with female to male ratio, F:M = 11:4, brain volume range: 1226777–1603417  mm^3^, median: 1396526  mm^3^) (Le Heron et al., 2018). CADASIL is a genetic form of small vessel disease caused by mutations within the NOTCH-3 gene. It is the most common heritable cause of stroke and vascular dementia in adults and it is characterised by extensive damage to white matter brain regions (Chabriat et al., 2009). These patients are younger than those with sporadic SVD, without confounding factors of co-existent neurodegenerative pathology (Attems and Jellinger, 2014). They therefore provide a model of pure SVD in which to investigate WMH (Charlton et al., 2006). Scanning was performed using the same scanner and acquisition parameters as the OXVASC dataset. Manual segmentation was not available for this dataset.

#### 2.1.4 Dataset 4: healthy controls (HC)

This dataset consists of 19 healthy controls, age-matched with the CADASIL subjects (described in Dataset 3) with age range 29–70 years, mean age 54.58 ± 11.25 years, median age 57 years, F:M = 6:13, brain volume range: 1313929–1679837  mm^3^, median: 1446443 mm^3^. Scanning was performed using the same scanner and acquisition parameters as datasets 2 and 3. Manual segmentation was not available for this dataset.

#### 2.1.5 Dataset 5: MICCAI WMH segmentation challenge dataset (MWSC)

The dataset consists of 60 subjects from three different sources (20 subjects each) provided as training sets for the challenge (http://wmh.isi.uu.nl/): UMC Utrecht - 3 T Philips Achieva, NUHS Singapore - 3 T S TrioTim, VU Amsterdam - 3 T GE Signa HDxt. Manual segmentations are available for all three datasets, with an additional exclusion label provided for other pathology. In the challenge, these masks with exclusion labels were ignored during performance evaluation. However, we included these masks as parts of non-lesion tissue, during the calculation of the performance metrics, for a more stringent evaluation in the presence of pathologies. The WMH volume ranges (excluding other pathologies) are 845–74991  mm^3^ (median: 26240 mm^3^) for UMC Utrecht, 786–61332 mm^3^ (median: 17795 mm^3^) for NUHS Singapore and 1522–43528 mm^3^ (median: 6015 mm^3^) for VU Amsterdam, and the brain volumes are 1257820–1844920 mm^3^ (median 1473389 mm^3^) for UMC Utrecht, 1147248–1532268 mm^3^ (median: 1351325  mm^3^) for NUHS Singapore and 1219614–1787321  mm^3^ (median: 1441201 mm^3^) for VU Amsterdam. For more details regarding MRI acquisition parameters and the image dimension details, refer http://wmh.isi.uu.nl/. Also, in the challenge the performance metrics were calculated on independent unseen test datasets. Since we did not participate in the challenge, we used publicly available training datasets for evaluation, using leave-one-out cross validation. Therefore, our results are not directly comparable with those published after the outcome of the challenge, as they are not relative to the unseen test data used in the challenge.

### 2.2 Image preprocessing

We skull stripped the images (used for intensity features) using FSL BET (Smith, 2002), followed by bias field correction using FSL FAST (Zhang et al., 2001). Diffusion-weighted images in datasets 2, 3, 4 were pre-processed as described in Zamboni et al. (2019) to extract mean diffusivity (MD) map, used as additional intensity feature. We registered the image modalities to the base modality (in our case, FLAIR for all datasets) using linear rigid-body registration with FSL FLIRT (Jenkinson and Smith, 2001). We also calculated the transformation between the subject’s native space and the MNI space, required by BIANCA for determining spatial features. For all the experiments presented in this work we used linear registration, calculated with FSL FLIRT, for estimating MNI coordinates as spatial features. However, we also tested the effect of using non-linear registration, obtaining negligible change in BIANCA performance (refer to supplementary material for more details).

### 2.3 BIANCA features and training options

Currently, default options in BIANCA are: spatial weighting (sw) ​= ​1, no patch, location of training points ​= ​any location for non-WMH training points, number of training points ​= ​Fixed ​+ ​equal with 2000 training points. For more details regarding the BIANCA options, refer Griffanti et al. (2016).

For NDGEN, we used FLAIR ​+ ​T1 as features. Other than the default options, we used the following non-default options: location of training points ​= ​no border, number of training points ​= ​Fixed ​+ ​unbalanced with 2000 lesion points and 10,000 non-lesion points. For OXVASC we used FLAIR ​+ ​T1 ​+ ​MD as features. The non-default options used were: sw ​= ​2, 3D patch with patch size of 3. For CADASIL and HC, we used the same features and BIANCA options used for OXVASC except spatial weighting. We excluded spatial features by setting sw ​= ​0 for these two datasets. For MWSC dataset, we trained BIANCA separately on the three datasets (UMC Utrecht, NUHS Singapore and VU Amsterdam), using the same features and BIANCA options used for NDGEN.

### 2.4 Use of population-level parametric lesion probability map (PPLPM)

Population-level parametric lesion probability maps (PPLPMs) describe the pattern of lesion distribution with respect to a specific parametric factor. Therefore, they could provide useful additional information to improve lesion segmentation. For our experiments in this work, we considered age as our parametric factor of interest, since a clear relationship has been established between age and WMH distribution (Simoni et al., 2012; Vannorsdall et al., 2009).

**Fig. 2:**
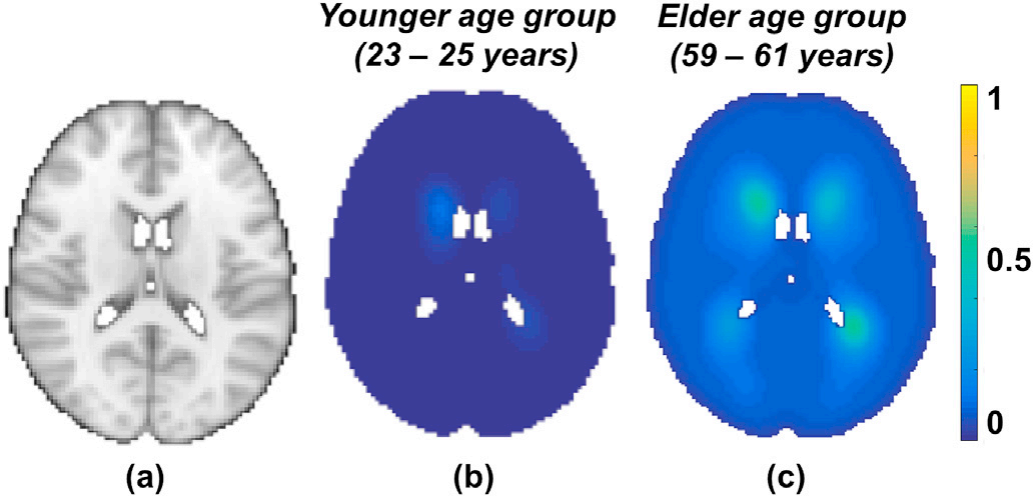
Parametric lesion probability maps at two example points in the parametric dimension, corresponding to two age groups (all the images are shown at z = 45 in MNI space, (a); in the younger age group (29–31 years, b) the lesion probability is very low throughout the brain, while in the older age group (59–61 years, c) the lesion probability is higher, especially in the periventricular regions.

We modelled the PPLPM with respect to age within a population consisting of 474 subjects, as described in our previous work (Sundaresan et al., 2019). Briefly, our Bayesian spline model takes binary lesion maps of individual subjects of the population as inputs and generates a 4D parametric lesion probability map, with age (grouped at intervals of 3 years) along the 4th dimension. The resulting parametric lesion probability map indicates the probability of lesion occurrence at a specific age group at each voxel. Fig. 2 shows the PPLPM at two example age groups, corresponding to 29–31 years and 59–61 years.

We used the PPLPM in BIANCA in two ways: either as an additional feature to the KNN classifier, or post-multiplying the PPLPM with the subject’s lesion probability map that is obtained using BIANCA with existing features.

For the first experiment, we implemented the PPLPM in BIANCA as follows: (1) we included the PPLPM (a 4D volume in the MNI space with the $4^{th}$ dimension representing different age groups) as an additional input, (2) we included age as an extra input in BIANCA and used it to select the appropriate 3D map from the PPLPM corresponding to the specific age group and (3) we transformed the 3D map from the MNI space to the subject’s native space non-linearly using FSL FNIRT (Andersson et al., 2007) before extracting its probability values as features to be used by the KNN classifier. For the second experiment, we multiplied the age-specific 3D map from PPLPM, transformed to the subject’s native space, with lesion probability map obtained from BIANCA (with the existing options and no additional features). We also tested the effect of the average 3D map across all age groups instead of the age-specific one.

We performed both experiments on the NDGEN dataset (keeping all the other options constant, as optimised in Griffanti et al., 2016 and specified in section *BIANCA features and training options*), and evaluated the performance with respect to the manual masks as described in section *Performance evaluation metrics*.

### 2.5 Comparison of alternative classifiers within BIANCA

Currently, BIANCA is based on the KNN algorithm. In this work we assessed the performance of four other classifiers: random forest (RF), neural networks (NN), support vector machine (SVM) and adaboost (AB). First we optimised the parameters for each classifier based on the area under the curve values from ROC curves, and then compared their results with KNN. Table - 1 provides the list of available parameters (from the python *scikit-learn* package) and the parameters that were considered for tuning in the optimisation step.

**Table 1:** Parameters considered for tuning different classifiers.

Classifier	Available main parameters and default values	Values of parameters considered for tuning
Random forest (RF)	No. of trees – 10 Criteria for splitting - MSE Maximum depth of trees - Till the leaves are pure Minimum no. of samples required for a split - 2 Minimum samples at each leaf node - 1 Maximum number of features for each tree - No. of features Minimum impurity measure at each split - $1\times 10^{-7}$ Boostraping done - True Out-of-bag error score - False	No. of trees - 20, 30, 40, 50, 100, 1000 (results for larger no. of trees shown in the supplementary material) Minimum samples at each leaf node - 200, 400, 500, 600, 800, 1000 Minimum impurity measure at each split - $1\times 10^{-7}$, $1\times 10^{-6}$, $1\times 10^{-5}$, $1\times 10^{-4}$, $1\times 10^{-3}$, $1\times 10^{-2}$ Maximum depth of trees - 25, 50, 100
Neural network (NN)	No. of neurons in a hidden layer - 100 Activation function - RELU Output activation function - Logistic sigmoid Solver - ADAM ($\beta_{1}=0.9,\beta_{2}=0.999,\varepsilon =1\times 10^{-8}$ ) No dropout L2 regularization, Alpha - $1\times 10^{-4}$Change in learning rate - Constant Initial learning rate - $1\times 10^{-3}$ Maximum no. of iterations - 200 Tolerance - $1\times 10^{-4}$ Momentum - 0.9 Fraction of samples for validation - 0.1	No. of neurons in a hidden layer - 100, 120, 150 Solver - LBFGS, ADAM Initial learning rate - $1\times 10^{-4}$, $1\times 10^{-3}$ Maximum no. of iterations - 1000, 1500, 1800 Tolerance - $1\times 10^{-4}$, $1\times 10^{-3}$, $1\times 10^{-2}$
Support vector machine (SVM)	Type of kernel - Radial basis function (RBF) *γ* - 1/(no.of features) Tolerance - $1\times 10^{-3}$ C-value - 1.0 Epsilon - 0.1 Maximum no. of iterations - Until convergence	*γ* - 0.001, 0.01, 0.1, 1.0, 10 C-value - 0.1, 1.0, 10 Maximum no. of iterations - 1000, 2000, 3000, 5000, 6000
Adaboost classifier (AB)	Base estimator - Decision Tree Regressor No. of trees in base estimator - 1 No. of estimators - 50 Learning rate - 1.0 Loss function - Linear	No. of trees in base estimator - 2 No. of estimators - 30, 40, 50 Learning rate - 0.5, 0.75, 1 Loss function - linear, exponential, square

**Fig. 3:**
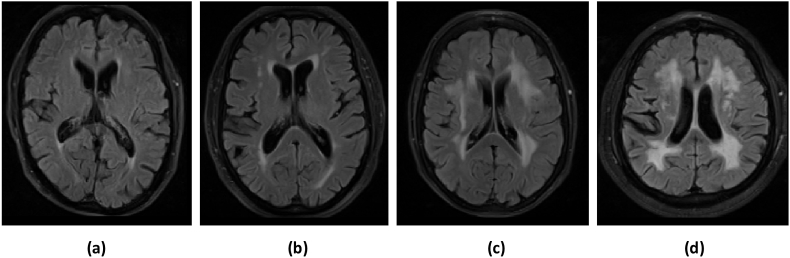
Examples of subjects with different WMH lesion loads used for parameter tuning of the classifiers: (a) low, (b) medium, (c) high and (d) very high lesion load. The manually segmented lesion volumes for low, medium, high and very high lesion loads are 5409 mm^3^, 21005  mm^3^, 50585  mm^3^ and 89259  mm^3^ respectively.

For initial parameter tuning, we selected four subjects from the NDGEN dataset with four different lesion loads: low, medium, high and very high (ranging from 5409 to 89259  mm^3^, see Fig. 3). After optimisation, we applied the four classifiers on the remaining 17 subjects from the NDGEN dataset (keeping all the other options constant, as specified in section *BIANCA features and training options*) and evaluated the performance with respect to the manual masks as specified in section *Performance evaluation metrics*. In order to test the consistency of the results across various cohorts, we tested the performance of the best performing alternative classifiers in the three cohorts from MWSC dataset (refer to the supplementary material for more details).

### 2.6 LOCally Adaptive Threshold Estimation (LOCATE)

In order to overcome the impact of spatial heterogeneity of lesion probabilities due to changes in lesion contrast, load and distribution on the final thresholded WMH map, we propose a method that determines spatially adaptive thresholds at different regions of the lesion probability map. LOCATE (LOCally Adaptive Threshold Estimation) takes as input the subject-level lesion probability map obtained from a lesion segmentation algorithm (in our case, BIANCA run with the options specified in section *BIANCA features and training options*) and estimates local thresholds in three steps by dividing the lesion probability map into sub-regions (using Voronoi tessellation), extracting local characteristics (features) within those sub-regions and estimating the optimal local threshold values based on the extracted features using a supervised learning method.

#### 2.6.1 Voronoi tessellation and feature extraction

**Fig. 4:**
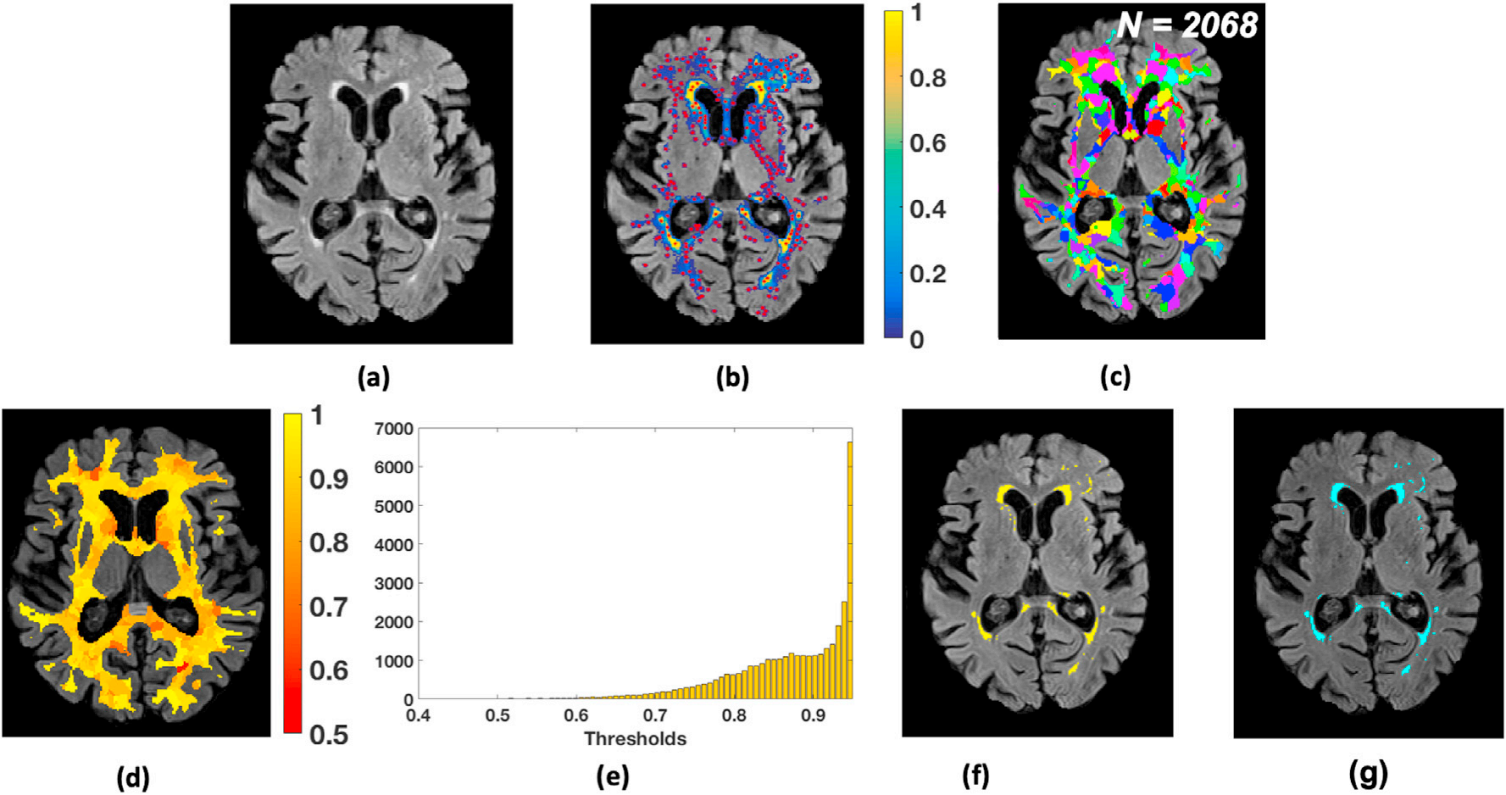
LOCally Adaptive Threshold Estimation (LOCATE). (a) FLAIR image; (b) detection of local maxima ($M_{i}$, red dots) on the smoothed lesion probability map (blue-yellow); (c) Voronoi spaces $V_{i}$ around local maxima points (indicated by random colours); (d) threshold map showing the local threshold values obtained from the regression model; (e) histogram of thresholds Th_opt; (f) final binary lesion map obtained by applying the thresholds (d) on the lesion probability map (b); (g) binary lesion map obtained by applying a global threshold of 0.9.

Firstly, we detected local maxima points $M_{i}$, where i=1...N on the lesion probability map to identify the plausible lesion locations (indicated by red dots in Fig. 4b). In order to avoid spurious local maxima points due to isolated voxels, we applied a small amount of spatial smoothing to the lesion probability map with a Gaussian filter, prior to local maxima detection. The FWHM size of the Gaussian kernel was empirically chosen to be 1.2 voxels (with standard deviation, σ=0.5 voxels).

We then tessellated the lesion probability map based on local maxima $M_{i}$ into *N* Voronoi polygons $V_{i}$ (Fig. 4c), around the maxima $M_{i}$. In order to ensure that our Voronoi polygons are within the region of interest (in our case, the brain white matter), we constrained the Voronoi polygons within a white matter mask obtained from a dilated and inverted CSF tissue segmentation, combined with other deep grey exclusion masks, as described in Griffanti et al. (2016).

Within each Voronoi polygon $V_{i}$, we applied different levels of thresholds (Th) from 0 to 0.9 with incremental steps of 0.05, and extracted the following features at each Th:

1. Mean greyscale intensity of the image used to identify the lesions (in our case, FLAIR) within the thresholded region.
2. Distance between the ventricles and the centre of gravity of the thresholded region. Lateral ventricles were segmented from T1 images as described in Griffanti et al., 2018 and the distance from the ventricle mask was calculated using the FSL command distancemap).
3. Volume of the thresholded region.

For tessellating the lesion map into sub-regions, we also experimented with simple linear iterative clustering (SLIC) and observed that LOCATE is robust to the method used for tessellation (refer to supplementary material for experiment details and results).

**Fig. 5:**
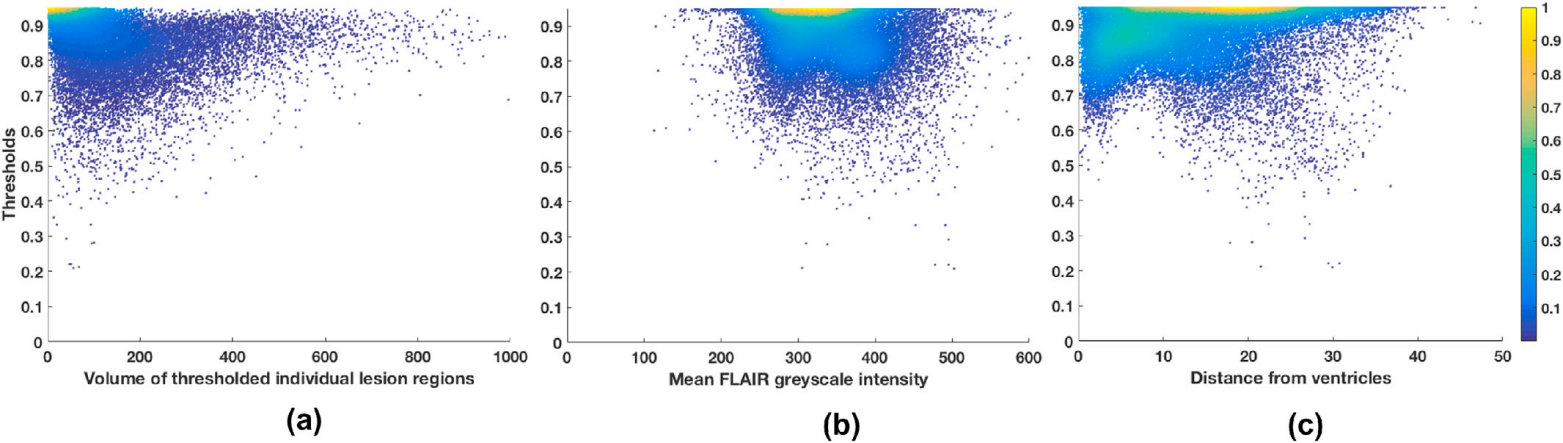
Features extracted from each Voronoi polygon plotted against the threshold values for 21 subjects from the NDGEN dataset: (a) volume of individual lesion region, (b) mean greyscale intensity and (c) distance of the centre of gravity from the ventricles mask. A simple straightforward relationship was not found between the features and the thresholds, indicating the need for a machine learning based method.

Due to lack of a simple relationship between the features and the thresholds (Fig. 5), we determined the optimal local threshold for each $V_{i}$ using a random forest (Breiman, 2001) regression model with 1000 trees and min leaf size of 5.

*Training phase:* For each $V_{i}$, among the set of thresholds Th we determined the highest value $Th_{max}$ at which we obtained the best similarity index with respect to the manually segmented binary lesion mask. We then trained the random forest regression model with the above features against $Th_{max}$.

*Testing phase:* We applied the trained regression model to get the optimal threshold Th_opt for each $V_{i}$ in the test images. Fig. 4d shows an instance of Th_opt map. Note that the periventricular region shows higher threshold values compared to the deep region, indicating the variation in lesion probabilities. Moreover, the histogram of thresholds Th_opt (Fig. 4e) obtained for an individual image shows a wider range of local threshold values depending on the local lesion characteristics and spatial distribution. As a final step, we thresholded the lesion probability map within each $V_{i}$ using the corresponding Th_opt to get the final binary lesion map (Fig. 4f).

#### 2.6.2 LOCATE evaluation and validation

We initially evaluated LOCATE using leave-one-out testing independently on the NDGEN, OXVASC and MWSC datasets on the outputs from BIANCA obtained using features and options specified in the section *BIANCA features and training options*. LOCATE performance was tested against the manual segmentation and against the results obtained using the optimal global threshold using the metrics described in section *Performance evaluation metrics*. In order to evaluate LOCATE performance in lesions with different characteristics, we additionally calculated the performance metrics in periventricular and deep lesions separately. We also performed ANOVA on the performance metrics to observe the main and interaction effects on the measures. We adopted the 10 mm distance rule: clusters within 10 mm distance from the ventricles were considered as periventricular lesions, otherwise as deep lesions (DeCarli et al., 2005). While there are other criteria available for identifying deep and periventricular lesions, the 10 mm distance rule is more suitable for automatic implementation, it is commonly used and agrees with the human rater, even in most of the confluent lesion cases (Griffanti et al., 2018).

We used CADASIL and HC datasets to further validate the robustness of LOCATE with respect to lesion load and the flexibility of LOCATE with respect to the training dataset. In fact, they represent two very different scenarios from the datasets tested so far. The subjects in the CADASIL dataset have a very different lesion pattern and, on average, a higher lesion load than the other datasets, while HC have negligible lesion loads. Moreover, since manual segmentation was not available for these datasets, we used the OXVASC dataset for training (both for obtaining the lesion probability map with KNN using the same features/options, and for LOCATE) since the images were acquired using the same MRI protocol. The output obtained with LOCATE on these datasets was qualitatively evaluated and quantitatively compared with the lesion mask obtained by applying the optimal global threshold (0.9), determined with leave-one-out on the training dataset. In addition, on the CADASIL subjects, we compared LOCATE output with the mask obtained by applying a lower global threshold (0.2). This threshold was empirically chosen (based on visual inspection of the lesion probability map) by an expert neurologist [CLH] as the optimal global threshold for this specific dataset. Using this comparative analysis, we explored the possibility of using LOCATE on different cohorts and various lesion loads, having a very different optimal global threshold from the pre-established value of 0.9. Also, by using CADASIL and HC datasets, we aimed to observe the effect of the difference in the lesion characteristics between patient group (CADASIL) and controls (HC) on thresholds obtained from LOCATE.

### 2.7 Performance evaluation metrics

We evaluated the lesion segmentation results using the following overlap measures and detection rates:

• Dice Similarity Index (SI): calculated as 2 × (true positive lesion voxels)/(true lesion voxels ​+ ​positive voxels). True lesion voxels refer to the lesion voxels in the manual segmentation and positive lesion voxels are the voxels labelled as lesions by the classifier. We used SI to generate SI plots at different thresholds and to perform paired t-tests between existing BIANCA and all the options used in the experiments described in the above sections.
• True positive rate (TPR): number of true positive lesion voxels divided by the number of true lesion voxels
• False positive rate (FPR): number of false positive lesion voxels (voxels incorrectly labelled as lesion) divided by the number of non-lesion voxels. We used TPR and FPR in order to plot ROC curves for evaluating the segmentation of existing BIANCA and all the options used in the experiments.
• Cluster-level true positive rates in deep and periventricular white matter: number of true positive lesions divided by total number of true lesions, calculated separately for deep and periventricular lesions. We used this metric to evaluate the robustness of LOCATE and to perform paired *t*-test between the global threshold and LOCATE results.

## 3 Results

### 3.1 Use of population-level parametric lesion probability map (PPLPM)

**Fig. 6:**
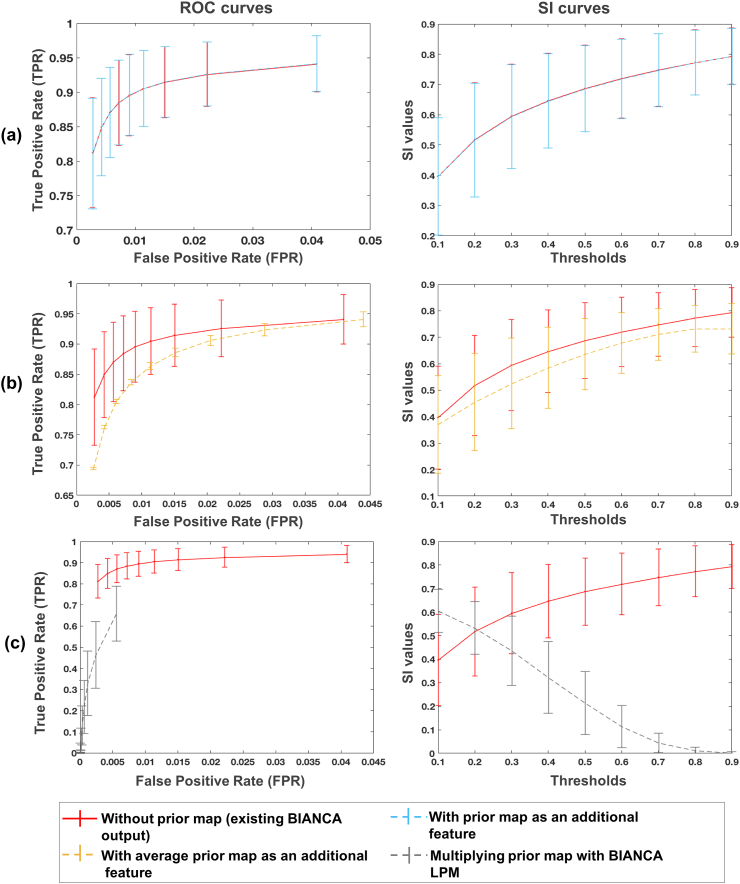
ROC (left) and SI (right) curves in the case of use of PPLPM when (a) the age-specific 3D map is added as an additional feature (light blue), (b) the average 3D map is added as an additional feature (yellow), and (c) when age-specific 3D map is post-multiplied with BIANCA lesion probability map (grey). The curves corresponding to the existing BIANCA results (Griffanti et al., 2016) are shown in red for comparison in all the three cases.

Fig. 6 shows the ROC curves and SI plots at different thresholds for our experiments using the PPLPM with respect to age on the NDGEN dataset. Using PPLPM as an additional feature does not improve the performance of BIANCA, although the use of the age-specific 3D map (Fig. 6a) still gives better results than the use of an average map across all age groups (Fig. 6b). When the PPLPM is post-multiplied with the subject’s lesion probability map (Fig. 6c), the TPR and SI values are very low, especially at higher thresholds.

The optimal threshold for the existing version of BIANCA for this dataset was found to be 0.9 (Griffanti et al., 2016). After adding the age-specific 3D map as a new feature, we still found 0.9 to be the optimal threshold for both age-specific and average cases, while for the post-multiplication case the optimal threshold was 0.1. The paired *t*-test showed significantly higher SI values for existing BIANCA (at the threshold of 0.9) compared to all cases using the parametric map (p = 0.02 for age-specific map as feature, threshold 0.9; p = 0.002 for average map as feature, threshold 0.9; p<0.001 for the post-multiplication, threshold 0.1).

### 3.2 Comparison of alternative classifiers within BIANCA

Table 2 lists the best performing set of parameters that were obtained for each classifier on the four subjects selected from the NDGEN dataset in the optimisation phase, when we tested using the area under the curve values for all possible combinations of the parameter values listed in Table 1.

**Table 2:** Best performing set of parameters for different classifiers.

Classifier	Best performing set of parameters
Random forest (RF)	No. of trees - 50
	Min. samples at each leaf node - 400
	Min. impurity measure at each split - $1\times 10^{-3}$
	Max. depth of trees - 25
Neural network (NN)	No. of neurons in a hidden layer - 100
	Solver - LBFGS, Initial learning rate - $1\times 10^{-3}$
	Max. no. of iterations - 1800
	Tolerance - $1\times 10^{-3}$
Support vector machine (SVM)	*γ* - 1.0
	C-value - 1.0
	Max. no. of iterations - 5000
Adaboost classifier (AB)	No. of trees in base estimator - 2
	No. of estimators - 30
	Learning rate - 1.0
	Loss function - exponential

**Fig. 7:**
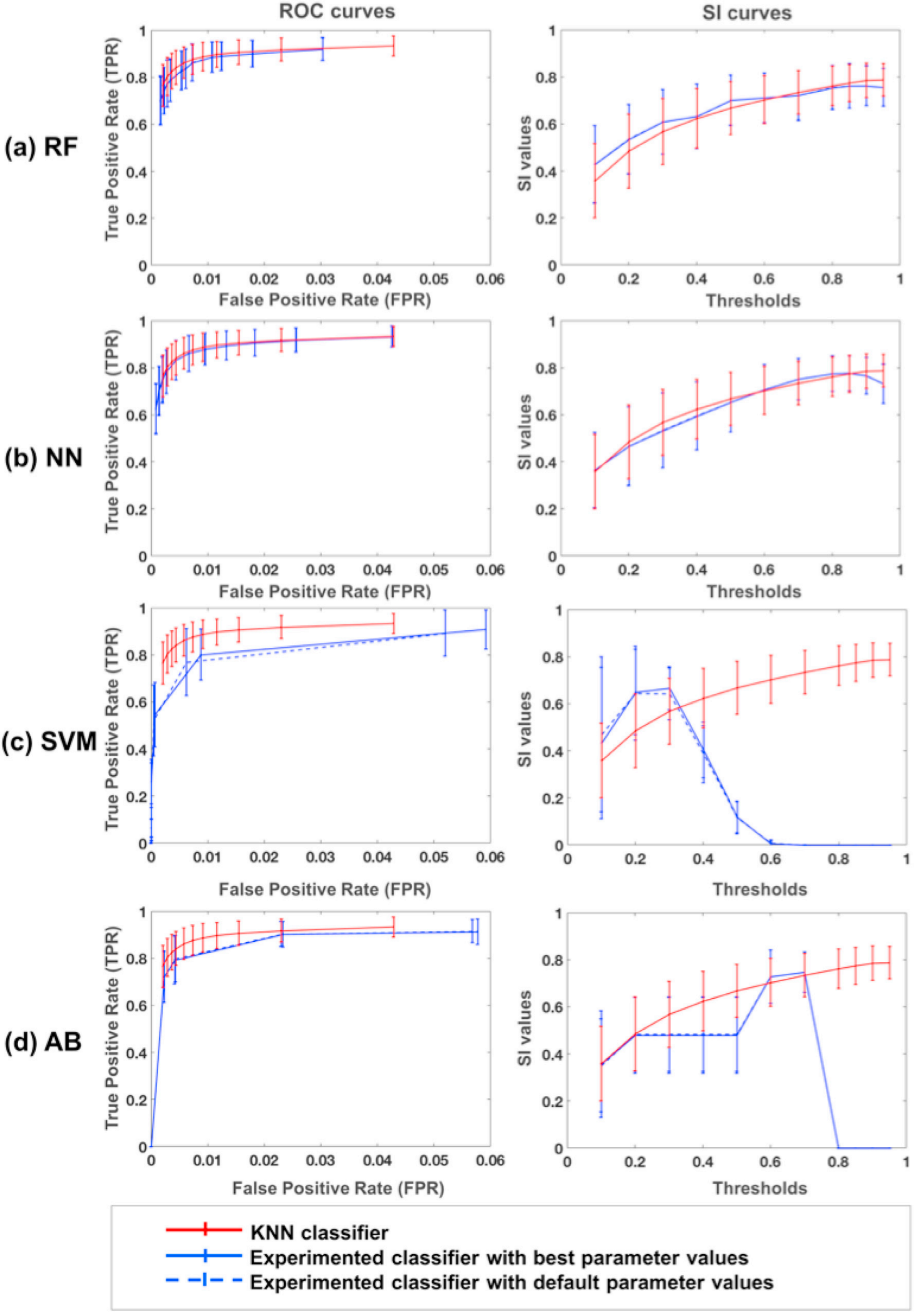
ROC curves (left) and SI curves (right) for alternative classifiers: (a) random forest (RF), (b) neural network (NN), (c) support vector machine (SVM) and (d) adaboost classifier (AB). Results are shown for each classifier’s best (blue solid line) and default parameters (blue dashed lines) along with the results for KNN classifier, currently used in BIANCA (red solid line).

Fig. 7 shows the ROC curves and SI plots for the four classifiers (RF, NN, SVM, AB) using the best and the default set of parameters, with respect to KNN, on the remaining 17 subjects from the NDGEN dataset. From the ROC curves and SI indices, overall KNN performs better than other classifiers, even for their best set of parameters and using the optimal threshold for each classifier. Paired *t*-test results showed that SI values from KNN (mean SI = 0.77 ± 0.10 at a threshold of 0.9) are significantly higher than those of RF (p = 0.02, mean SI for RF = 0.76 ± 0.10 at a threshold of 0.9), SVM (p < 0.001, mean SI for SVM = 0.75 ± 0.10 at a threshold of 0.3) and AB (p < 0.001, mean SI for AB = 0.65 ± 0.12 at a threshold of 0.7), while SI values using NN are not significantly different from KNN (p = 0.32, mean SI for NN = 0.75 ± 0.09 at a threshold of 0.8). Similar results were obtained on the MWSC datasets: on NUHS Singapore and UMC Utrecht cohorts KNN performed significantly better than NN and AB, and similar to RF. On VU Amsterdam cohort, SI values obtained using the KNN classifier were not significantly different from those obtained with all alternative classifiers. For more details on these results and relative plots, refer to the supplementary material.

### 3.3 LOCally Adaptive Threshold Estimation (LOCATE)

**Fig. 8:**
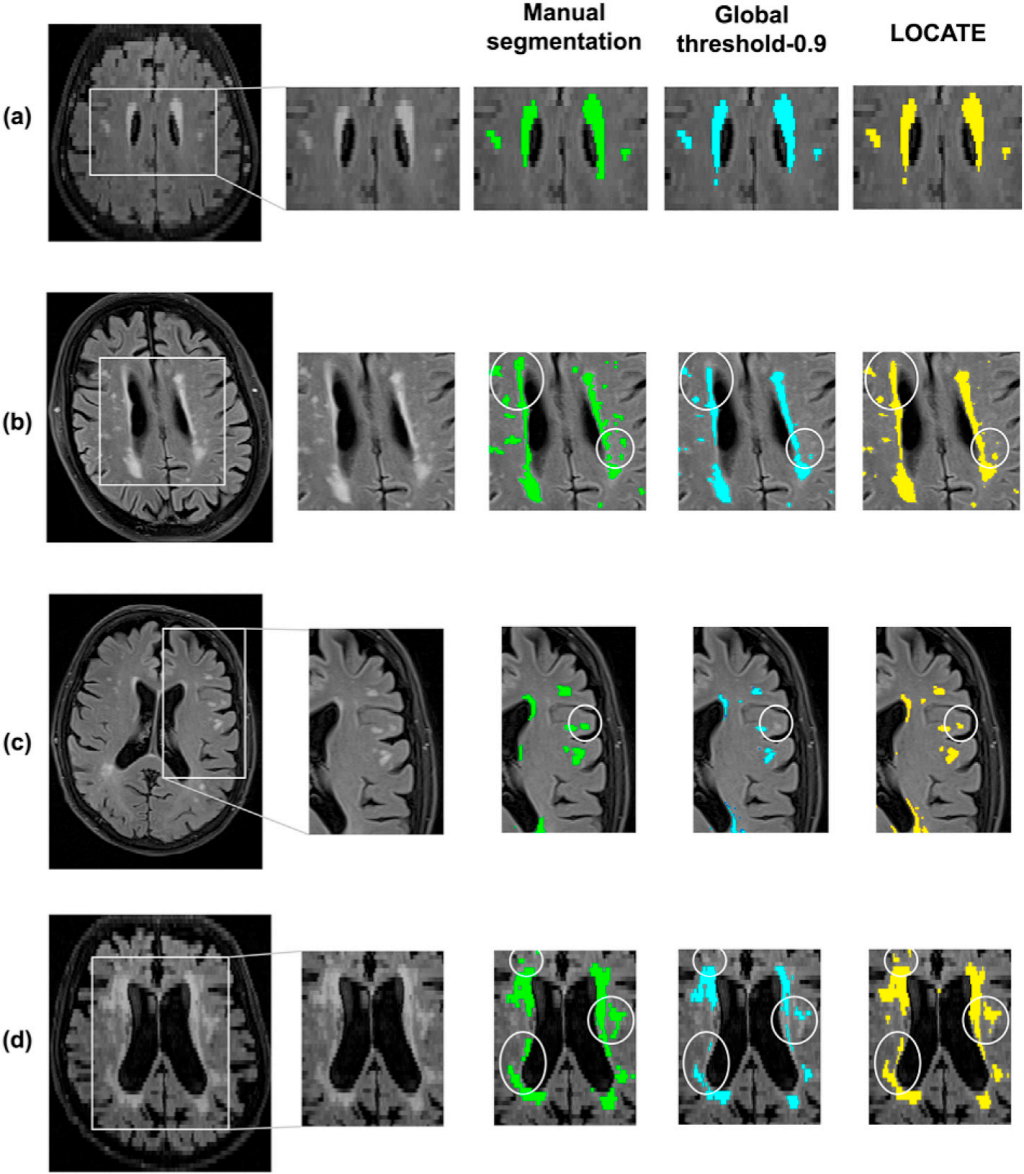
Example results of LOCATE for (a) low, (b) medium, (c) high and (d) very high lesion load (a,d from the OXVASC; b,c from the NDGEN datasets). Manual segmentation (green) is shown along with outputs of global thresholding at 0.9 (light blue) and LOCATE (yellow). LOCATE provides better segmentation in deep (c, d) and periventricular white matter lesions (a, b, d) when compared to global thresholding.

**Fig. 9:**
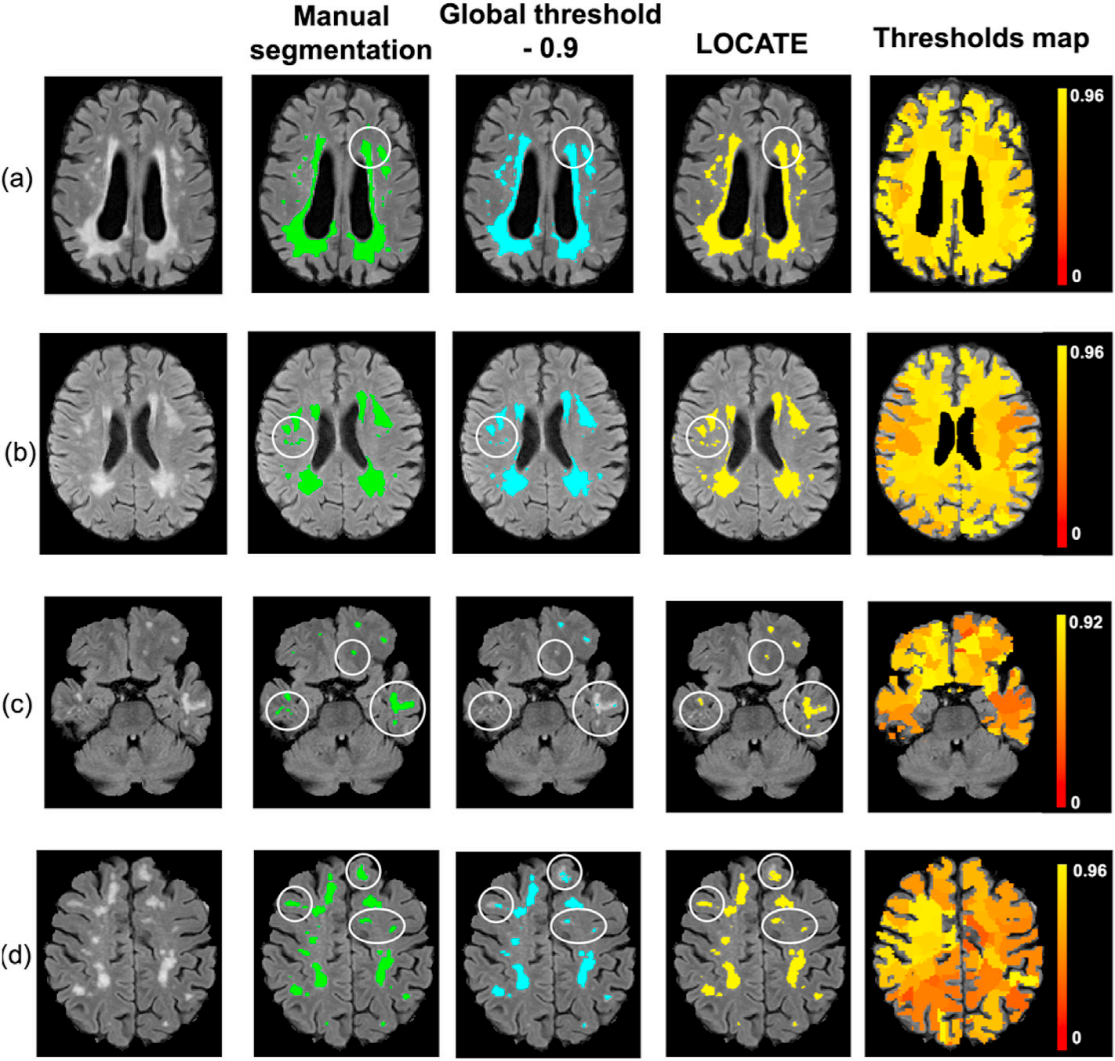
Example results of BIANCA with locate (yellow) and global thresholding (blue), compared to the manual segmentation (green). The corresponding threshold maps are shown in red-yellow (note the heterogeneity of lesion probabilities shown in the brain white matter).

Fig. 8, Fig. 9 illustrate examples of BIANCA results with LOCATE outputs compared to outputs with global threshold and the manual segmentation in various datasets. LOCATE detected more deep lesions (Fig. 8c and d and Fig. 9b, and frontal region of d) and segmented periventricular lesions better (Fig. 8a, b, d and Fig. 9a) with respect to the manual segmentation. The additional lesions detected by LOCATE with respect to manual segmentation in 8b, c, d did not represent false positives as they were labelled as lesions by the manual segmentation in adjacent slices. In MWSC datasets LOCATE segments the lesions in the temporal regions with better accuracy (Fig. 9c) compared to global threshold, which misses a few well-defined lesions in that region.

**Fig. 10:**
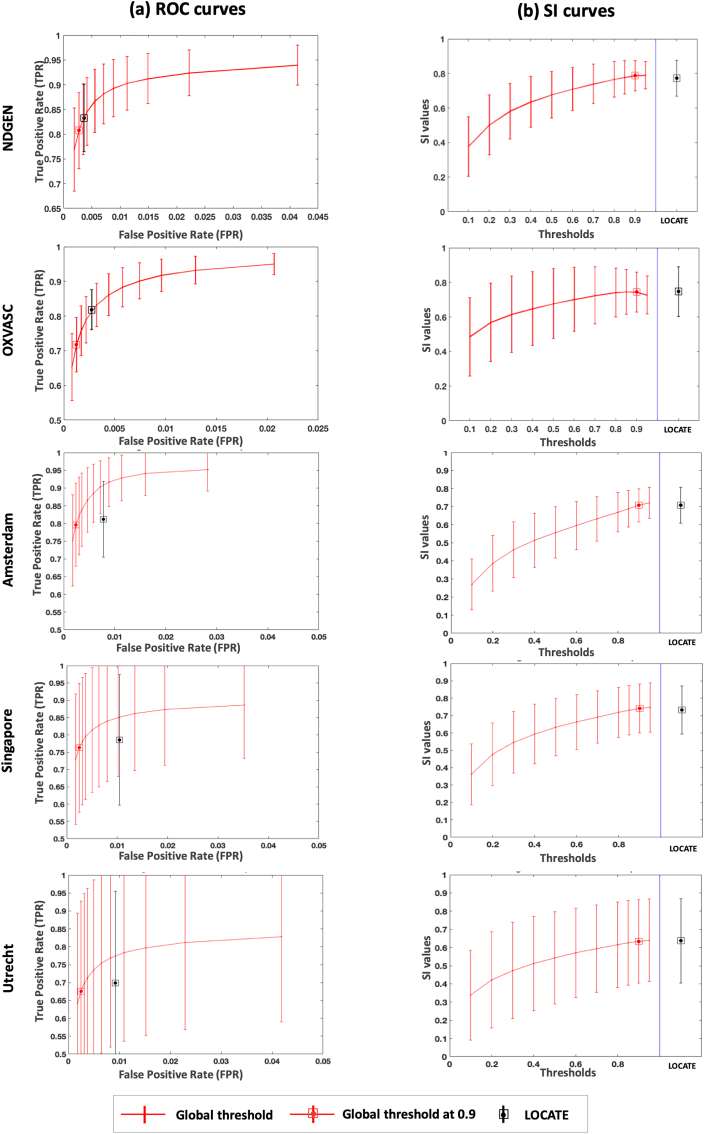
Comparison of ROC curves, SI plots for LOCATE and global thresholding. ROC curves (a), SI indices (b) for NDGEN, OXVASC and MWSC (VU Amsterdam, NUHS Singapore, UMC Utrecht) datasets.

Fig. 10 shows the ROC curves and SI plots for BIANCA at various global thresholds and using LOCATE for NDGEN, OXVASC and MWSC datasets. The SI values obtained with LOCATE were not significantly different according to the paired *t*-test to those obtained with global thresholding values of 0.9 for NDGEN (LOCATE SI = 0.77 ± 0.10; global threshold SI = 0.77 ± 0.08, p=0.94), OXVASC (LOCATE SI = 0.75 ± 0.14; global threshold SI = 0.74 ± 0.11, p=0.94) and MWSC (UMC Utrecht: LOCATE SI = 0.64 ± 0.23, global threshold SI = 0.63 ± 0.22, p=0.29, NUHS Singapore: LOCATE SI = 0.73 ± 0.13, global threshold SI = 0.74 ± 0.14, p=0.93, VU Amsterdam LOCATE SI = 0.70 ± 0.09, global threshold SI = 0.70 ± 0.09, p=0.46) datasets. However, in terms of detection rates, LOCATE gave an increase in voxel-wise TPR (0.03 for the NDGEN and 0.10 for the OXVASC) with a negligible increase in FPR (0.001 for the NDGEN and 0.002 for the OXVASC) compared to the global threshold of 0.9. In case of the MWSC, the voxel-wise TPR showed negligible difference between LOCATE and global thresholding results, despite a small increase in FPR. It is worth noting that for MWSC datasets, we obtained the above results on the publicly available training datasets (using leave-one-out cross validation), while in the challenge the evaluation was done on the held out test dataset and hence the performance of our method could change depending on the dataset characteristics.

**Fig. 11:**
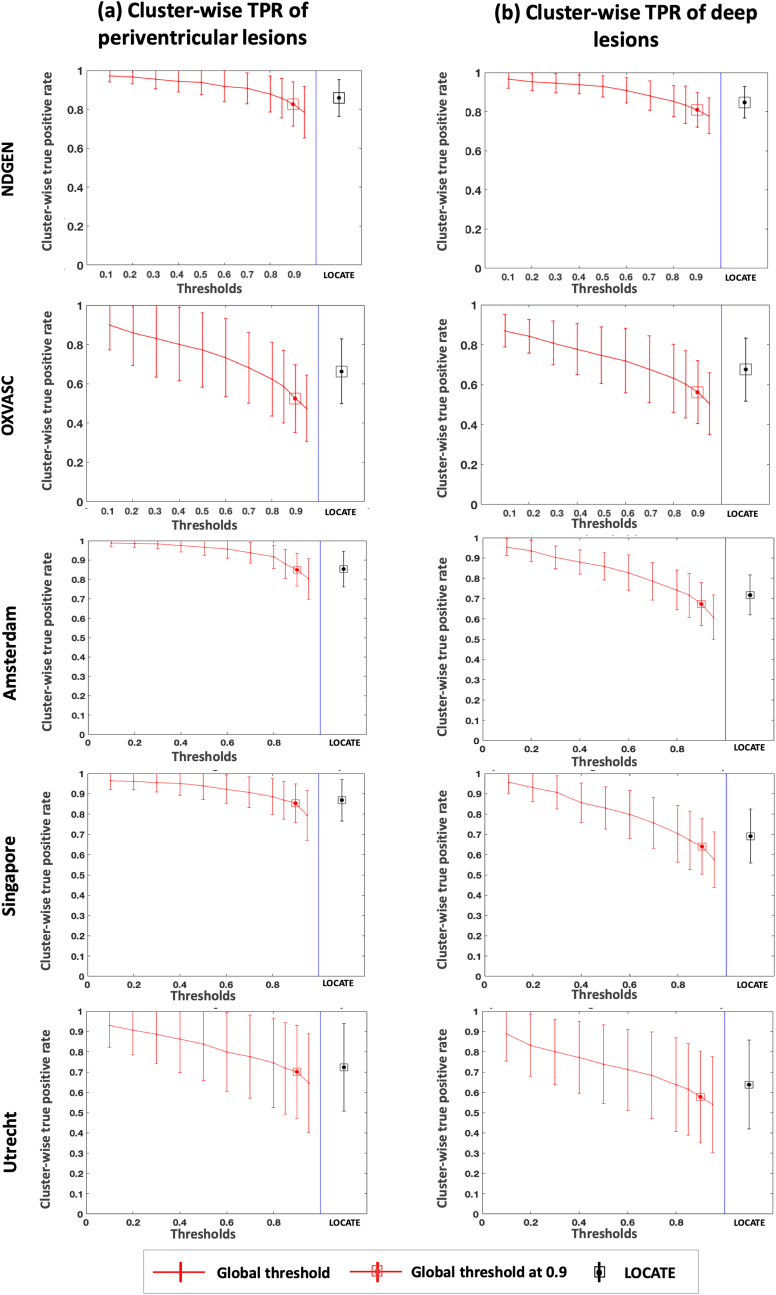
Comparison of cluster-wise true positive rates in periventricular and deep region for LOCATE and global thresholding. Cluster-wise true positive rates in periventricular (a) and deep (b) regions shown for NDGEN, OXVASC and MWSC (VU Amsterdam, NUHS Singapore, UMC Utrecht) datasets.

Fig. 11 shows the cluster-wise TPR plots for NDGEN, OXVASC and 3 cohorts of MWSC respectively. We performed 2-way repeated measures ANOVA considering region (deep and periventricular) and method (LOCATE and Global thresholding at 0.9) as independent factors and cluster-wise TPR as the dependent measure. We determined the main effect of region and method, along with the effect of their interaction, on cluster-wise TPR values. Table 3 reports the descriptive statistics and ANOVA results for comparison of cluster-wise TPR values. We observed a significant main effect of method for all datasets, with cluster-wise TPR always higher for LOCATE than global thresholding. The main effect of region was significant only for MWSC cohorts: cluster-wise TPR was not significantly different between periventricular and deep regions in NDGEN and OXVASC, while it was significantly higher in periventricular than deep regions for MWSC cohorts. In these cohorts we also observed a significant interaction effect: the increase in cluster-wise TPR using LOCATE with respect to global threshold was higher in the deep regions compared to periventricular regions. For the similar comparative analysis and ANOVA results on other measures such as TPR, FPR and SI values for the above datasets, refer to the supplementary material.

**Table 3:** Descriptive statistics and ANOVA results for comparison of cluster-wise TPR values for global threshold of 0.9 and LOCATE in periventricular and deep regions for MWSC datasets.

			NDGEN	OXVASC	VU Amsterdam	NUHS Singapore	UMC Utrecht
Descriptive statistics	Deep region	LOCATE	0.85 ± 0.08	0.67 ± 0.16	0.71 ± 0.09	0.69 ± 0.13	0.64 ± 0.22
		Global 0.9	0.80 ± 0.09	0.56 ± 0.16	0.67 ± 0.10	0.64 ± 0.14	0.58 ± 0.23
	Peri-venticular region	LOCATE	0.86 ± 0.09	0.66 ± 0.16	0.86 ± 0.08	0.87 ± 0.09	0.72 ± 0.21
		Global 0.9	0.83 ± 0.11	0.52 ± 0.18	0.85 ± 0.08	0.85 ± 0.09	0.70 ± 0.23
ANOVA	Main effect of region (deep/PV regions)		F (1,19) = 0.22 p = 0.64 $\eta_{p}^{2}$ = 0.011	F (1,16) = 0.44 p = 0.51 $\eta_{p}^{2}$ = 0.027	**F(1,19)** = **48.7** **p**<**0.001** $\eta_{p}^{2}$ = **0.719**	**F(1,19)** = **31.5** **p**<**0.001** $\eta_{p}^{2}$ = **0.623**	**F(1,19)** = **9.30** **p** = **0.007** $\eta_{p}^{2}$ = **0.327**
	Main effect of method (LOCATE/Global 0.9)		**F(1,19)** = **38.3** **p**<**0.001** $\eta_{p}^{2}$ = **0.668**	**F(1,16)** = **83.5** **p**<**0.001** $\eta_{p}^{2}$ = **0.839**	**F(1,19)** = **31.9** **p**<**0.001** $\eta_{p}^{2}$ = **0.627**	**F(1,19)** = **50.1** **p**<**0.001** $\eta_{p}^{2}$ = **0.725**	**F(1,19)** = **34.6** **p**<**0.001** $\eta_{p}^{2}$ = **0.646**
	Interaction (region * method)		F (1,19) = 0.22 p = 0.65 $\eta_{p}^{2}$ = 0.011	F (1,16) = 0.69 p = 0.42 $\eta_{p}^{2}$ = 0.041	**F(1,19)** = **17.7** **p**<**0.001** $\eta_{p}^{2}$ = **0.482**	**F(1,19)** = **16.30** **p** = **0.001** $\eta_{p}^{2}$ = **0.462**	**F(1,19)** = **10.80** **p** = **0.004** $\eta_{p}^{2}$ = **0.362**

**Fig. 12:**
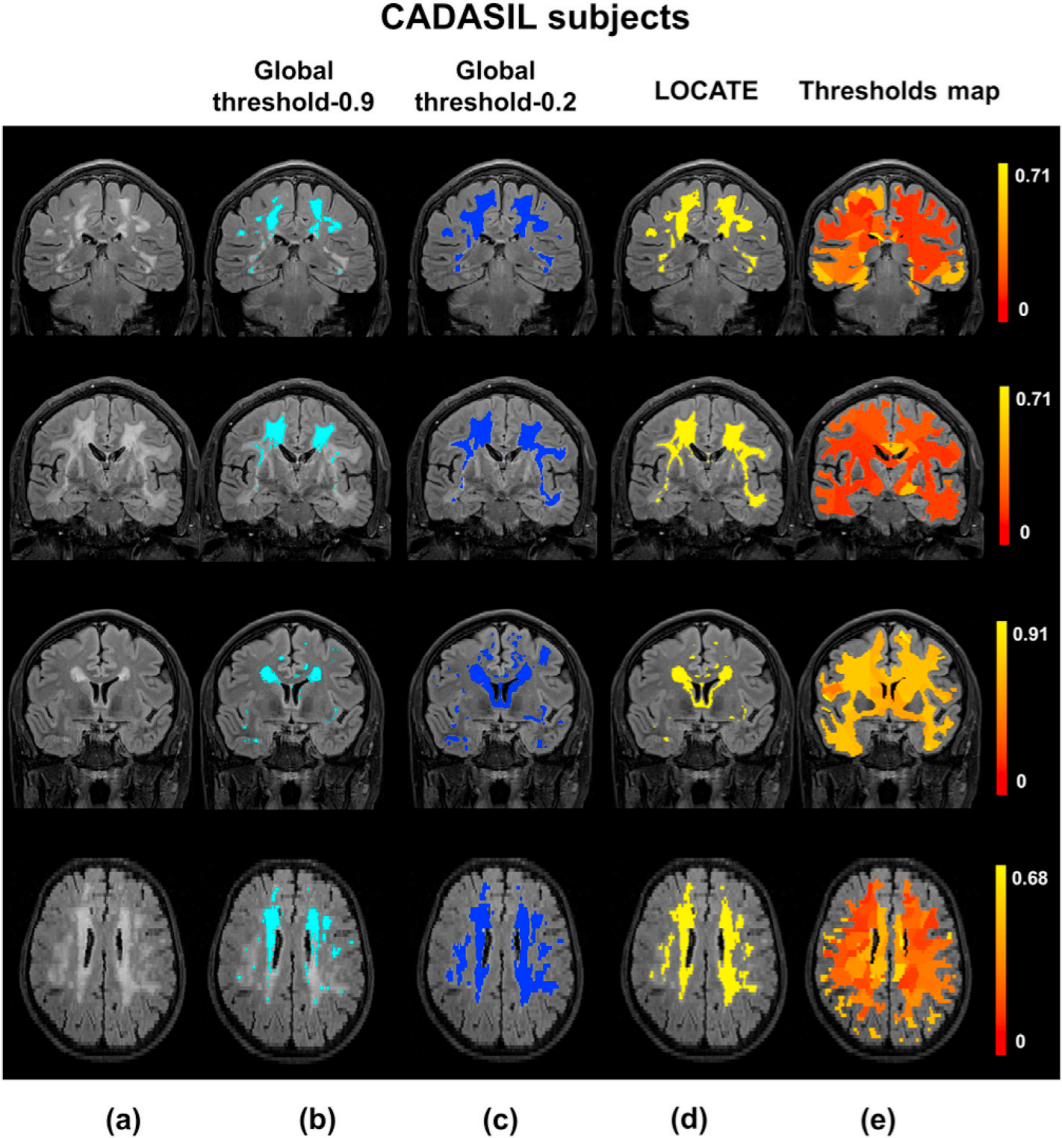
Example results of LOCATE on the CADASIL data. (a) FLAIR image, (b) global thresholding at 0.9 (light blue), (c) global thresholding at 0.2 (dark blue), (d) LOCATE results (yellow) and (e) threshold maps obtained from LOCATE (red-yellow). Note that the threshold maps shows the heterogeneity in the lesions probabilities in various regions of the white matter.

**Fig. 13:**
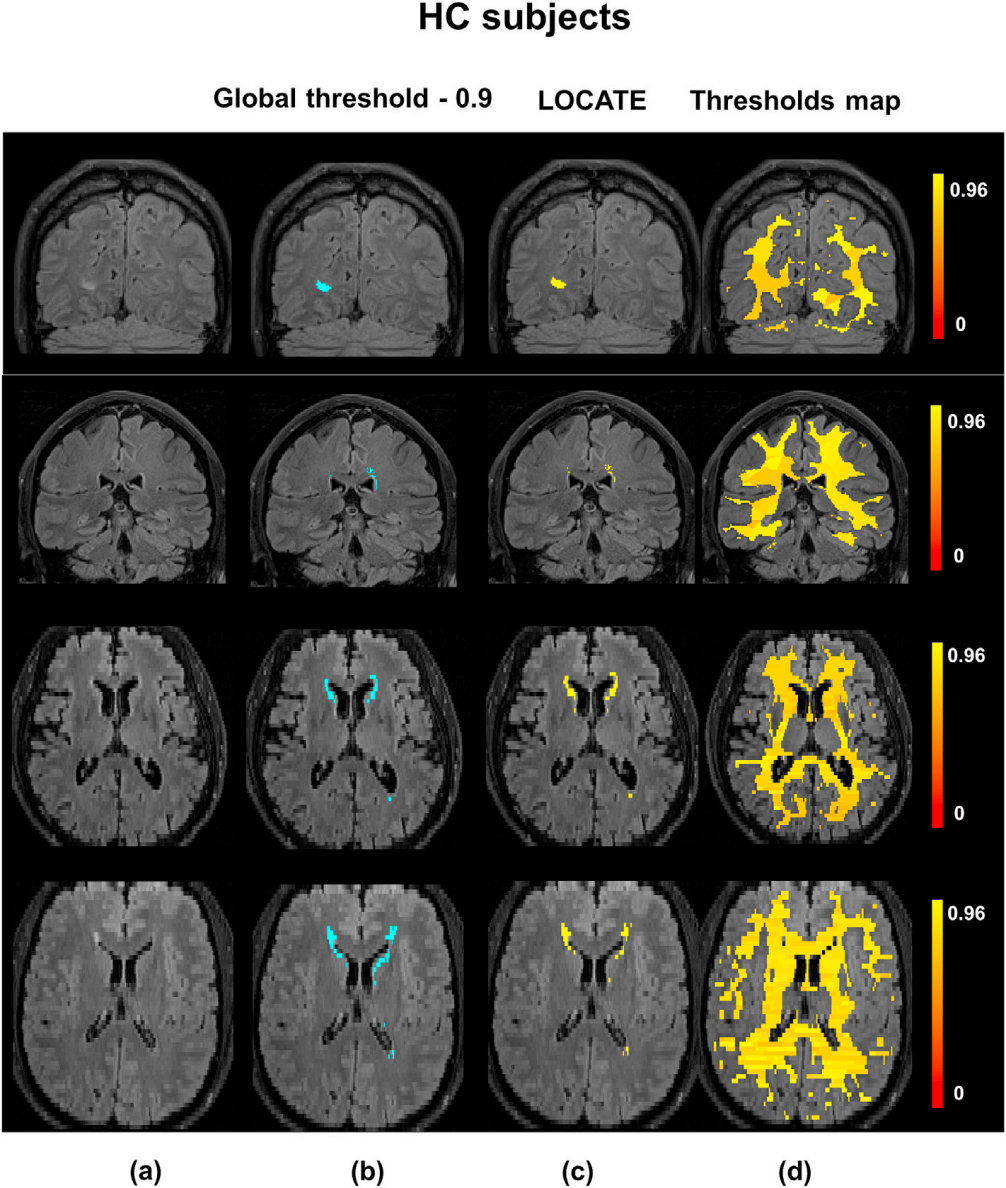
Example results of LOCATE on the HC data. (a) FLAIR image, (b) global thresholding at 0.9 (light blue), (c) LOCATE results (yellow) and (d) threshold maps obtained from LOCATE (red-yellow). Note that the threshold maps shows the heterogeneity in the lesions probabilities in various regions of white matter.

Fig. 12 illustrates the results of lesion segmentation by applying LOCATE on the CADASIL dataset. Since CADASIL patients have different lesion characteristics compared to those in the OXVASC dataset (different lesion location and very high lesion loads), applying the global threshold of 0.9 that was determined in the training phase on the OXVASC dataset yields poorly segmented binary lesion maps (Fig. 12b). The global threshold visually determined as optimal (by an expert neurologist CLH) was 0.2 (Fig. 12c), however this leads to increased false positives in some cases, as shown in the third case of Fig. 12c. LOCATE provides a much better segmentation and detects the lesions in the temporal lobe, typical of the pathology (Fig. 12d). A similar comparison of LOCATE results with those of global thresholding at 0.9 on the HC dataset is shown in Fig. 13.

When the binary lesion maps obtained using the optimal global threshold value of 0.2 is used as the reference segmentation, the paired *t*-test results show that the SI values obtained for CADASIL with LOCATE (SI = 0.79 ± 0.01) are significantly higher than those obtained using a global threshold at 0.9 (SI = 0.57 ± 0.10, p<0.001). For CADASIL, using LOCATE increases the voxel-wise TPR by 0.48 for a constant FPR of 0.00.

**Fig. 14:**
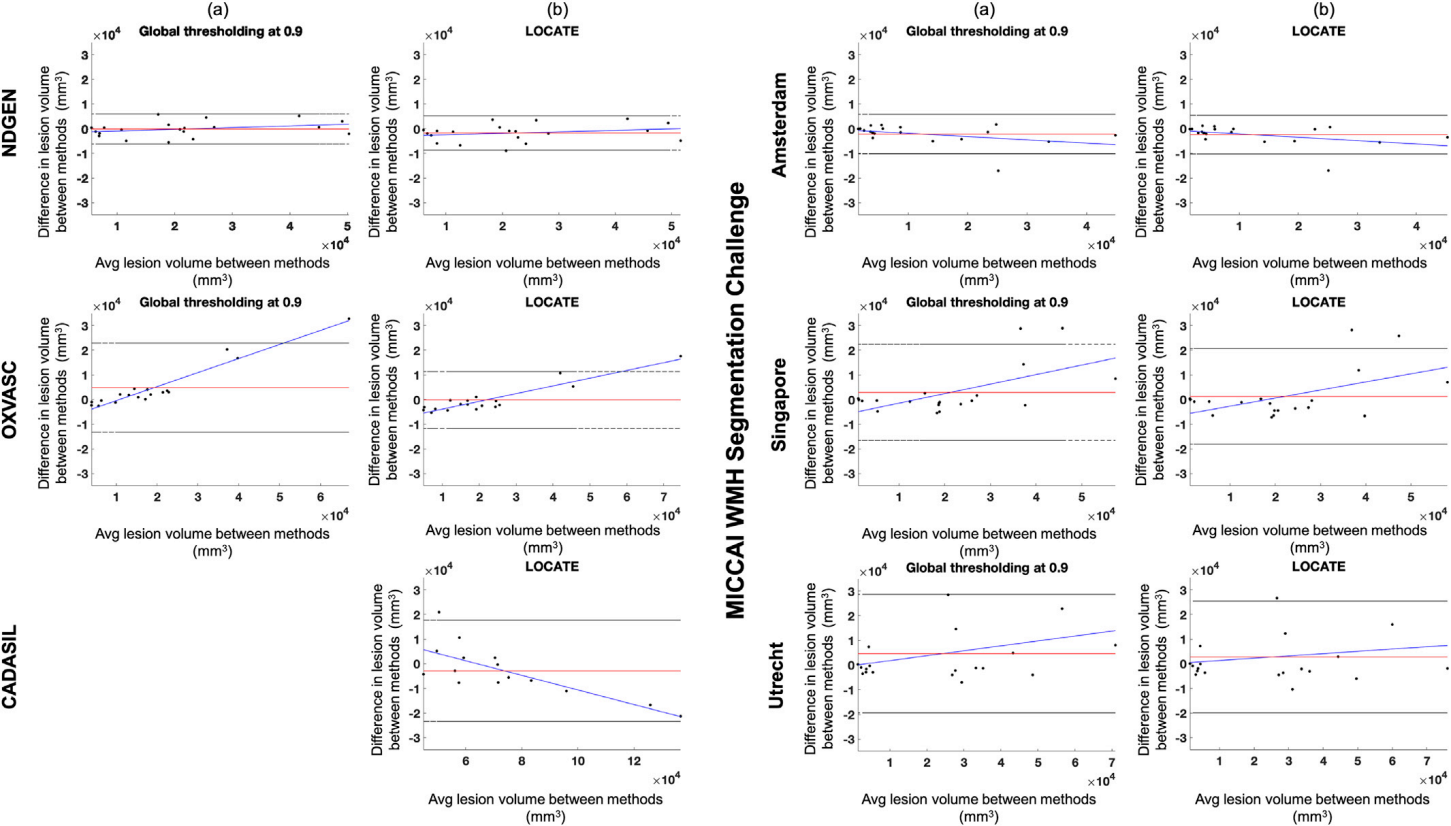
Bland-Altman plot of WMH volumes on results obtained from reference segmentation and global thresholding (a) and reference segmentation and LOCATE (b).

The Bland-Altman plots in Fig. 14 show the agreement in lesion volumes for global threshold (a) and LOCATE (b) with respect to the reference segmentation for the above datasets. From the figure, it can be seen that there is not much scope for improvement in NDGEN and VU Amsterdam, however LOCATE shows improvement for OXVASC, NUHS Singapore and UMC Utrecht both in terms of slope and limits of agreement.

**Fig. 15:**
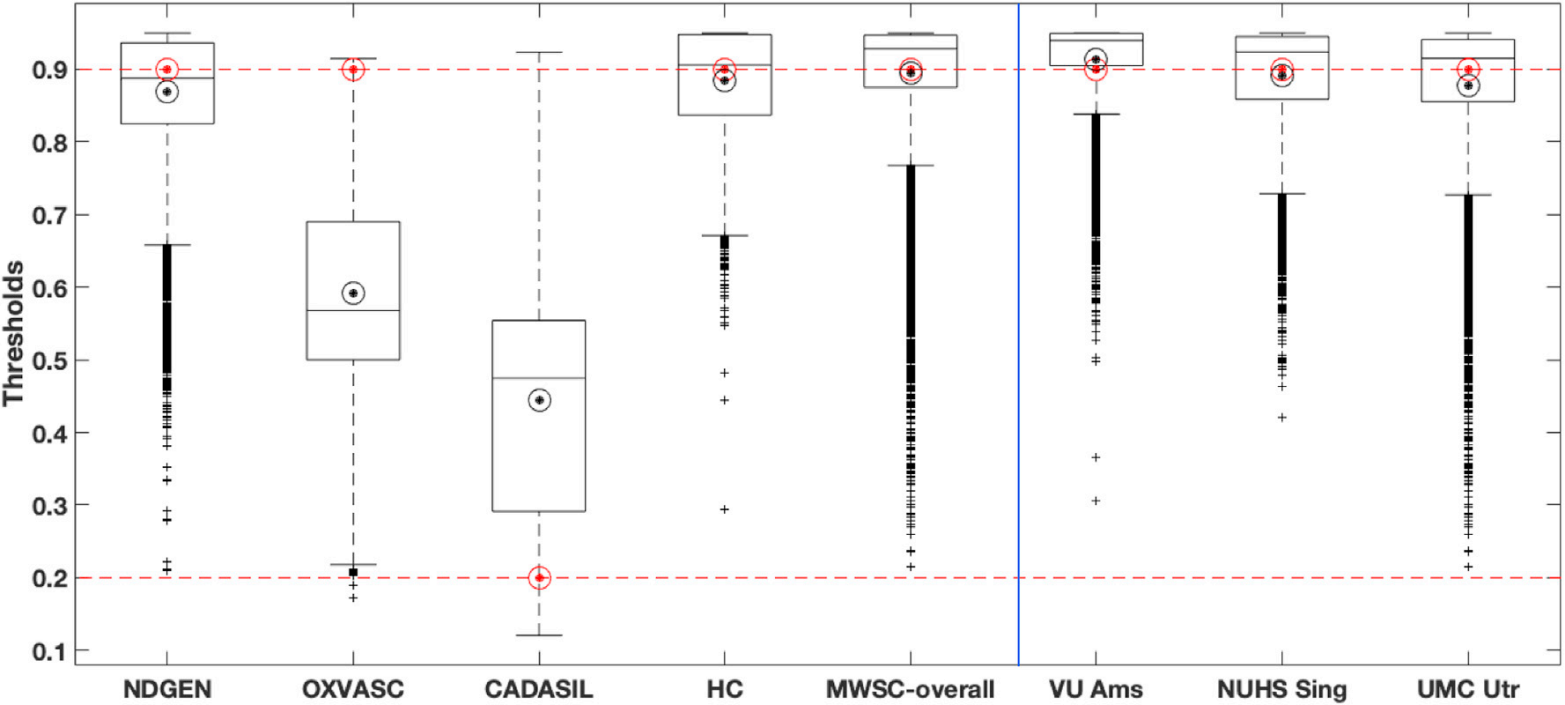
Boxplots of local thresholds obtained from the random forest regression model in LOCATE. The optimal global thresholds determined manually are shown in red circles. The mean values of local thresholds obtained from LOCATE are shown in black circles.

To summarise the effect of LOCATE on different datasets, Fig. 15 shows boxplots of local threshold values determined by LOCATE for the five datasets (also individually for the three MWSC datasets), along with the optimal global threshold values for each dataset. The median threshold values of NDGEN, MWSC and HC datasets are higher than other two datasets, especially the CADASIL dataset. LOCATE assigned higher thresholds for datasets with lower lesion load (HC and NDGEN, which contains 30% of healthy subjects) and lower thresholds for datasets with higher lesion load (OXVASC and especially CADASIL). This is also observable from the thresholded maps shown in Figs. 12e and 13d. Interestingly, the medians of LOCATE thresholds for the OXVASC and CADASIL datasets are also quite different from their corresponding optimal global thresholds and the ranges of thresholds obtained from LOCATE are wider (excluding the outliers), when compared to the other datasets. This could be due to the different sequence characteristics (e.g. lower resolution) and pathological characteristics (high amount of small vessel disease) of both these datasets with respect to the others.

In line with the results of the paired *t*-test, the largest improvement in SI corresponds to the largest difference between the LOCATE threshold and the global threshold. In the case of the HC dataset, the median of local thresholds obtained from LOCATE is very close to the optimal threshold of 0.9, thus showing only a slight improvement in the lesion segmentation. This is also evident from Fig. 13, where LOCATE results appear very similar to the results of global thresholding at 0.9 (Fig. 13b and 13c).

## 4 Discussion

In this work, we studied the effect of single-subject and population-level heterogeneity in amount, location and characteristics of WMH on the performance of BIANCA, with the aim of improving both the classification and the thresholding steps. At the classification step, we analysed the effect on the subject-level lesion probability maps of using population-level lesion probability map (PPLPM, with age as factor of interest) and of using alternative classifiers, which have been previously used for this task in the literature. For thresholding the lesion probability map, as an alternative to selecting a global threshold, we proposed LOCATE, a method to determine local thresholds that are sensitive to spatial differences in the lesion probabilities, giving more accurate binary lesion masks.

We observed the effect of using the PPLPM and found that post-multiplying the age-specific 3D map with the lesion probability map performs worse than adding the PPLPM as an additional feature. This is because PPLPM indicates the likelihood of finding a lesion at a given location in a population, informed primarily by the anatomy of disease signs. On the other hand, the subject-level probability map reflects the probability of identifying a lesion versus the normal tissue and is primarily determined by the intensity contrasts and noise in the image. For instance, in our previous work (Sundaresan et al., 2019) we showed that in PPLPM the lesion probabilities typically increase more in the periventricular WM than in the deep WM in the elder population (which is in line with the existing literature Simoni et al., 2012; Van Den Heuvel et al., 2004; Sabayan et al., 2015). Therefore, post-multiplying the PPLPM with the subject-level lesion probability map can bias the subject-level lesion probabilities more towards the periventricular lesions, irrespective of the local spatial characteristics in the images from the individual subject. However, when we provide the PPLPM as an additional feature, the classifier assigns sensible lesion probability values taking into account the PPLPM as well as the image intensity contrasts for the different modalities and the spatial coordinates. Particularly, we observed that adding age-specific map as an additional feature gives better results than the average map, since the former models the lesion probabilities for the subject’s age group specifically and hence is more accurate. Overall we observed that, at present, using the PPLPM does not improve significantly the performance of BIANCA (also evident from the p-values of our *t*-test results). Hence, our current recommendation is that PPLPM (with age as parameter of interest) is not necessary for improving BIANCA segmentation.

However, the way this option will be implemented in a future release of BIANCA is quite flexible and will allow the user to use any 4D PPLPM with respect to any other factor of interest, which could improve the segmentation in their specific dataset. Also, it is worth noting that before using this option, the user needs to ensure if the demographics of the test dataset are comparable to those of the population used to model the PPLPM.

The results on the comparison between KNN and other classifiers showed that KNN provided better segmentation. For the same set of features (excluding the PPLPM), we observed that SVM and AB classifiers provided significantly lower SI than KNN, especially at higher thresholds. On the other hand, SI values for RF and NN followed a similar trend to KNN with relatively similar SI values (Fig. 7). In the NDGEN dataset, SI values for RF were still significantly lower than KNN, while NN gave SI values that were not significantly different from KNN. In the MWSC dataset, SI values obtained using KNN did not differ significantly from those of RF in all cohorts and from those of NN in the VU Amsterdam cohort (refer to the supplementary material for the details). Moreover, for a given FPR, KNN has a higher TPR compared to any other classifier, indicating that KNN detects more true lesions than other classifiers. However, since NN and RF have the potential to perform as well as KNN, they will be included as alternative classifiers in a future release of BIANCA. At this point, it is worth noting that irrespective of the classifier used, there is a certain degree of uncertainty associated with the manual segmentation - they are an approximation to the real ground truth, meaning that very fine discrimination may reflect errors in the manual segmentation rather than errors in the automated classification results.

Regarding the improvements at the thresholding step with LOCATE, the initial leave-one-subject-out results of LOCATE on NDGEN, OXVASC and MWSC datasets showed improvement in BIANCA segmentation for all datasets. LOCATE detected more true positive lesions, especially in the deep white matter, and provided better delineation of the lesions with respect to the manual segmentation as indicated by the improved SI values and the visual results shown in Fig. 8, Fig. 9. Since BIANCA is currently not optimised for segmentation of (juxta)cortical, cerebellar and subcortical lesions, we could not further evaluate LOCATE performance in these areas, since they are not included in BIANCA output.

LOCATE provided results comparable with global threshold, irrespective of changes in the acquisition protocols and image characteristics in MWSC dataset. Top ranking methods for MICCAI WMH segmentation challenge are based on Deep Stack Networks and Ensemble Learning (Li et al., 2018), Multi-dimensional Gated Recurrent Units (Andermatt et al., 2016) and a modified location sensitive deep convolutional neural network (Berseth, 2017). The methods were ranked based on the set of performance metrics described in http://wmh.isi.uu.nl/, including SI values (Dice) and cluster-wise true positive rate (Sensitivity of individual lesions). We achieved an average SI value of 0.70 for both LOCATE and global thresholding, while the top ranking methods achieved SI values of 0.80, 0.78 and 0.77. Also, we achieved cluster-wise true positive rate of 0.75 with LOCATE and 0.71 with global thresholding, while the top ranking methods achieved 0.87, 0.83 and 0.73. Therefore, LOCATE gives a better cluster-wise TPR when compared to global threshold and the third ranking method in the challenge. However, a direct comparison would not be possible since the results of the top ranking methods were determined on the independent test datasets, while our results were obtained on leave-one-out validation on the MWSC training datasets that are publicly available. Also, we retained the regions corresponding to other pathologies for a more stringent evaluation, while these regions were ignored during evaluation in the MICCAI challenge.

Our validation of LOCATE on CADASIL and HC datasets proves that LOCATE is robust with respect to variations in lesion load and location without the need to retrain both BIANCA and LOCATE. In fact, LOCATE can be trained on any data having the same modalities, acquired with the same sequence to the test dataset. For instance, we used the OXVASC dataset to train LOCATE for testing it on the CADASIL dataset since both datasets were acquired with the same sequences. The pattern of lesions for CADASIL subjects differs from that of vascular subjects in the OXVASC dataset, as CADASIL subjects, in addition to the normal distribution of WMH seen in SVD, will typically have lesions within the external capsule and anterior temporal lobes (Chabriat et al., 2009). Due to this variability in lesion characteristics and pattern, training BIANCA on the OXVASC dataset yields very low lesion probabilities in the temporal regions. Reflecting these lower lesion probabilities, the thresholds map from LOCATE (Fig. 12) shows much lower threshold values, especially in the temporal regions in the CADASIL images. Therefore LOCATE yields much better segmentation performance (SI = 0.79) compared to a global threshold of 0.9 (SI = 0.57), when the lesion probability map thresholded at 0.2 was considered as the reference segmentation. Moreover, LOCATE results on CADASIL showed a substantial increase of 0.48 in TPR without an increase in FPR, due to the detection of more lesions in the temporal region. This indicates that LOCATE takes into account different lesion patterns and hence can be used in different pathologies. Also, compared with the approaches proposed by Ling et al. (2018), LOCATE overcomes the need for manually determining thresholds for individual subjects on the CADASIL dataset, while still providing similar SI values and comparable limits of agreement with the gold standard on Bland-Altman plot (Fig. 14 versus Ling et al., 2018, Fig. 7, col. 3).

The threshold maps obtained from LOCATE for the HC dataset show higher thresholds when compared with the CADASIL dataset. This is due to the fact that HC have mostly periventricular lesions (that are common in healthy ageing) that are usually assigned higher probabilities. Hence by using higher threshold values, LOCATE gives results that appear visually similar to the global thresholding at 0.9, detecting mostly periventricular lesions. While the threshold maps from LOCATE (Fig. 12, Fig. 13) provide information regarding the spatial heterogeneity of lesion probabilities, boxplots of thresholds shown in Fig. 15 show the overall characteristics of the datasets. The improvement in segmentation performance is more prominent in the OXVASC and CADASIL datasets when compared to the NDGEN, MWSC and HC datasets. This is reflected in their corresponding optimal global thresholds being very different from the median of their LOCATE thresholds. LOCATE results on the five datasets, especially the HC dataset (which has negligible lesion load compared with the CADASIL subjects) show that LOCATE is more adaptive to the variation in the global lesion load in addition to the spatial heterogeneity in lesion probabilities, compared to global thresholding (shown in Fig. 12, Fig. 13).

Regarding the number of subjects needed for training, we used a minimal training set of 18 subjects from OXVASC to get good performance on both OXVASC (on 17 subjects, with leave-one-out) and CADASIL. Hence we would recommend using 15–20 subjects for training LOCATE. However, it is worth noting that the number of training subjects needed to achieve good results can vary across datasets (for more details on the optimal number of training subjects, refer to Griffanti et al., 2016). For this reason, we advise checking the segmentation accuracy on each dataset, comparing the output with the manual masks whenever available and, if needed, increasing the number of subjects included in the training or consider re-training on the specific dataset. So far, evaluation of LOCATE has been performed on relatively small datasets (containing 15 20 subjects) which limits the power of our results. Also, the experiments done so far show that LOCATE is generalizable when trained with dataset-specific images or when using a training dataset generated with images acquired with the same scanner and sequences. We are currently working towards improving robustness across scanners and sequences. In its current Matlab implementation, LOCATE running time to determine a binary lesion map from an individual lesion probability map is approximately 15 min per subject, when run on an iMac with a 2.9 GHz Intel Core i5processor.

LOCATE will be included as an additional option for generating binary lesion maps, as an alternative to the faster, but less accurate, global thresholding, in a future release of BIANCA. The MATLAB implementation of LOCATE is currently available to use (see https://fsl.fmrib.ox.ac.uk/fsl/fslwiki/BIANCA/Userguide under post-processing section for more details).

A potential future development of LOCATE could be to use the estimated thresholds to normalise the lesion probabilities locally in order to obtain a more uniform lesion probability map that is robust to the diffuse nature of the lesions in different areas of the brain. Another interesting future direction for BIANCA could be to investigate the amount and distribution of lesions in various cerebral lobes, similar to our analysis of lesions in periventricular and deep regions. Also, various deep learning networks have been shown to perform better than the conventional machine learning based classifiers for similar problems. Hence in future work, we will explore various convolutional neural network models for more accurate segmentation of WMH.

## Funding

This work was supported by the Engineering and Physical Sciences Research Council (EPSRC) and Medical Research Council (MRC) [grant number EP/L016052/1]. The Oxford Vascular Study is funded by the National Institute for Health Research (NIHR) Oxford Biomedical Research Centre (BRC), Wellcome Trust, Wolfson Foundation, the British Heart Foundation and the European Union’s Horizon 2020 programme (grant 666881, SVDs@target). The Wellcome Centre for Integrative Neuroimaging is supported by core funding from the Wellcome Trust (203139/Z/16/Z). Professor PMR is in receipt of a NIHR Senior Investigator award. The views expressed are those of the author(s) and not necessarily those of the NHS, the NIHR or the Department of Health. VS is supported by the Oxford India Centre for Sustainable Development, Somerville College, University of Oxford. CLH was funded by a University of Oxford Christopher Welch Scholarship in Biological Sciences, a University of Oxford Clarendon Scholarship and a Green Templeton College Partnership award (GAF1415_CB2_ MSD_758342). MH was funded by a grant from the Wellcome Trust (206330/Z/17/Z) and the NIHR Oxford Biomedical Research Centre. MJ and GZ are supported by the National Institute for Health Research (NIHR) Oxford Biomedical Research Centre (BRC). LG is supported by the Monument Trust Discovery Award from Parkinsons UK (Oxford Parkinsons Disease Centre), the MRC Dementias Platform UK and the National Institute for Health Research (NIHR) Oxford Biomedical Research Centre (BRC).
