# Supplementary material for: Automated lesion segmentation with BIANCA: Impact of population-level features, classification algorithm and locally adaptive thresholding
Source: Neuroimage. 2019 Nov 15;202:116056. doi: 10.1016/j.neuroimage.2019.116056 (PMC6996003; doi:10.1016/j.neuroimage.2019.116056)
Supplement: Multimedia component 1 [file mmc1.pdf]

Supplementary material - Automated lesion segmentation with BIANCA: impact of population-level features, classification algorithm and locally adaptive thresholding

Comparison of BIANCA performance using linear and non-linear registration for estimating spatial features

Currently, in BIANCA the all the intensity features are calculated in native space and a linear transformation is required as input to extract the spatial features (spatial coordinates in MNI space). However, a non-linear registration is in general more suitable for registering single-subject images to MNI, especially in pathological brains. Hence, we performed experiments with non-linear registration for getting spatial coordinates in MNI space, to observe the improvement in BIANCA performance.

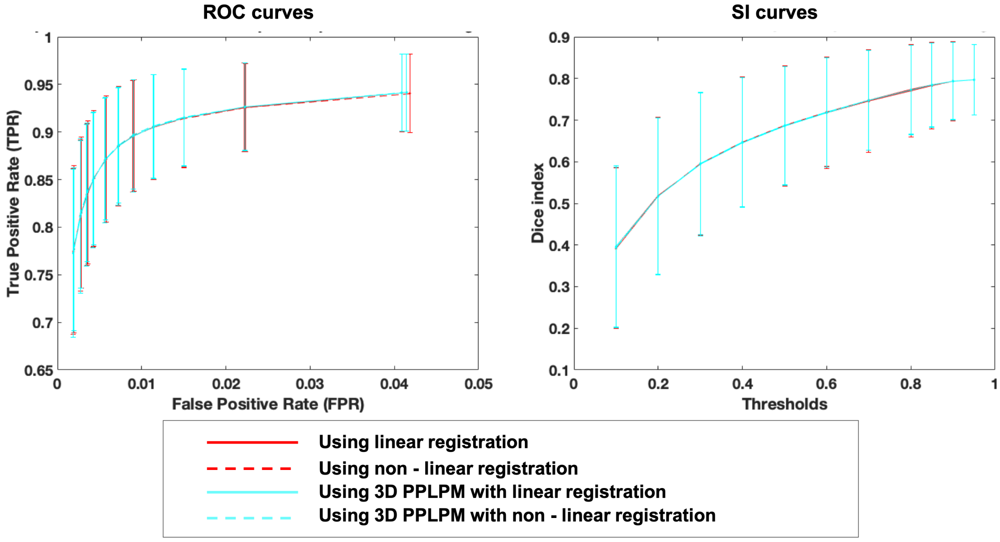

Figure S1: ROC (left) and SI (right) curves for the cases with linear and non-linear registration for extracting spatial features, with and without using PPLPM.

Figure S1 shows the ROC curves and SI curves for this experiment on images of neurodegenerative cases (dataset 1), which would contain more cases of atrophic

brains and therefore benefit the most from a non-linear registration. It can be seen from the figure that performing non-linear registration, with or without using PPLPM, gave negligible difference in the BIANCA performance. This is probably due to the fact that the classifier in BIANCA takes other features into account in addition to the spatial coordinates and that the voxel-level prediction is largely influenced by the intensity features.

Since the use of non-linear registration could still be beneficial in more extreme cases, in a future release of BIANCA users will be able to provide non-linear warp fields as an alternative to the linear transformation matrix for getting spatial coordinates.

Alternative classifiers: additional experiments

BIANCA performance using random forest (RF) classifier with larger number of trees

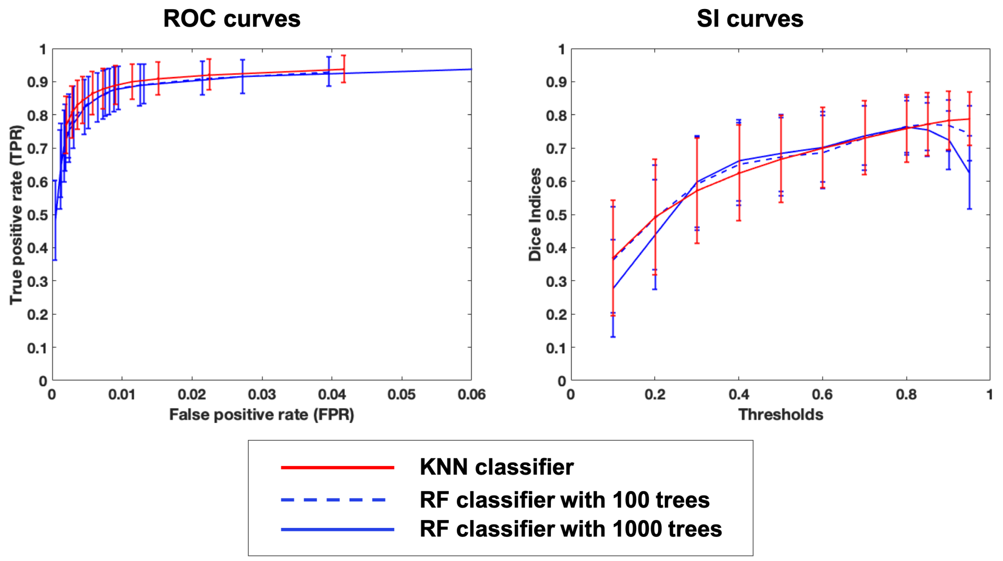

Figure S2: ROC curves (left) and SI curves (right) for random forest classifier for 100 trees (blue dashed lines) and 1000 trees (blue solid lines) against KNN classifier currently used in BIANCA (red solid lines).

For the classifiers experiment specified in section *Comparison of alternative classifiers within BIANCA* to observe the effect of various classifiers, we did an initial preliminary test on the random forest classifier, with wider range of trees (20, 30, 40, 50, 100, 1000). We then narrowed it down, since we observed the best performance for lower number of trees. For the random forest classifier, higher number of trees

has been shown to provide better results for various applications. Hence, we have provided the results using higher number of trees.

In particular, we show the ROC curves and SI curves for 100 and 1000 trees in figure S2. As it can be observed in the figure, the SI values are higher at lower thresholds, but they decrease at higher thresholds when increasing the number of trees.

Comparison of alternative best performing classifiers with KNN in MWSC dataset

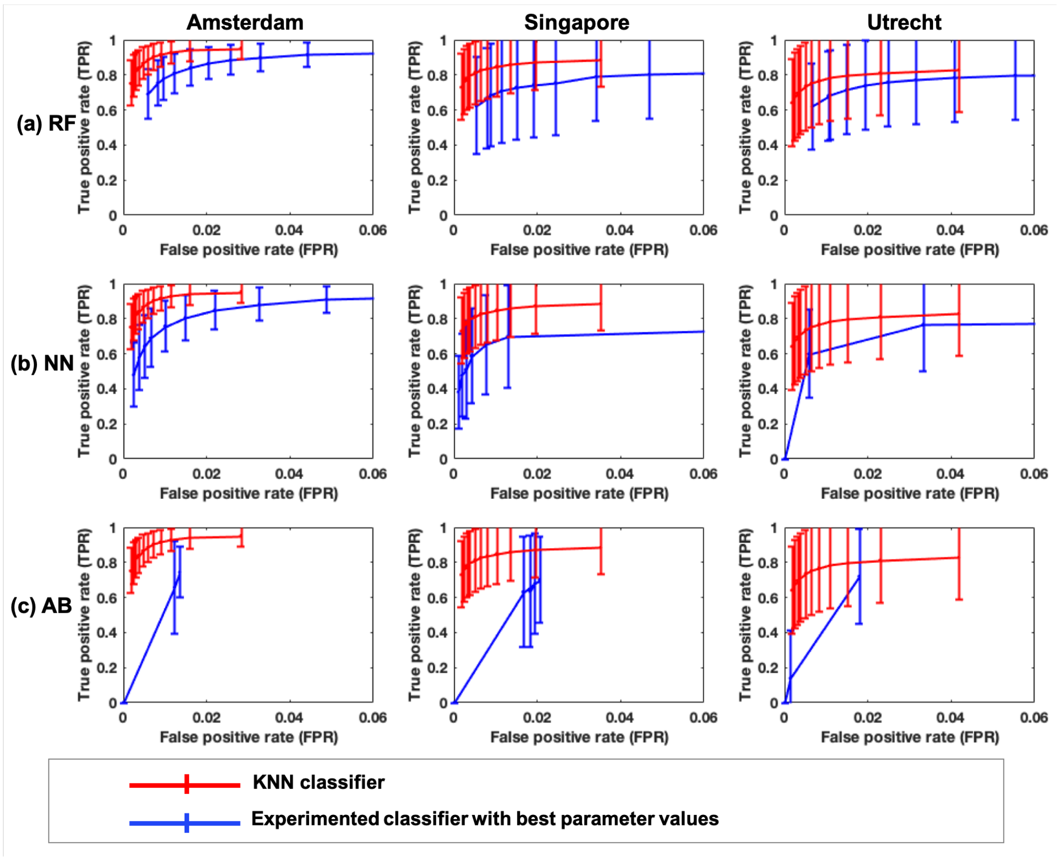

Figure S3: ROC curves for alternative classifiers: (a) random forest (RF), (b) neural network (NN) and (c) adaboost classifier (AB) for VU Amsterdam, NUHS Singapore and UMC Utrecht cohorts. Results are shown for each classifier's best parameters (blue solid line) along with the results for KNN classifier, currently used in BIANCA.

In NDGEN dataset, the SI values for KNN were significantly higher than RF, SVM and AB, and non-significantly different from NN. To test the consistency of this difference across cohorts, we applied the best performing alternative classifiers (RF, NN and AB) with their best parameters (listed in Table 2 in the main manuscript)

on the MWSC dataset, since it consists of data from three different cohorts: VU Amsterdam, NUHS Singapore and UMC Utrecht. Figures S3 and S4 show the ROC and SI curves for RF, NN and AB against KNN on the 3 MWSC cohorts. Comparing these figures with Figure 7 of the main manuscript, the trend of ROC and SI curves for all the classifiers across all cohorts remain similar to those of NDGEN, although the NN classifier gives slightly lower SI values than the KNN classifier in the Singapore and Utrecht cohorts.

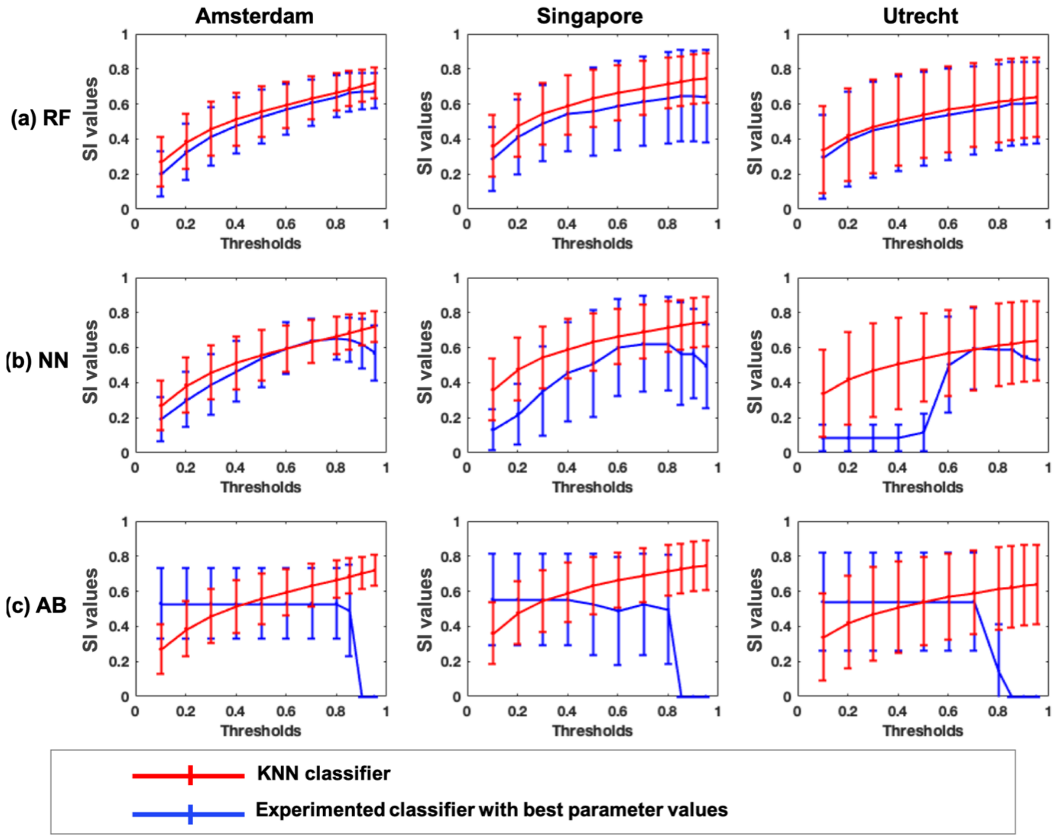

Figure S4: SI curves for alternative classifiers: (a) random forest (RF), (b) neural network (NN) and (c) adaboost classifier (AB). Results are shown for each classifier's best parameters (blue solid line) along with the results for KNN classifier, currently used in BIANCA.

The paired t-test results on the SI values of KNN and alternative classifiers (shown in Figure S4) showed the following results:

- On the VU Amsterdam cohort, SI values obtained using the KNN classifier are not significantly different from those obtained from alternative classifiers (RF: mean SI = 0.67 ± 0.10 at a threshold of 0.9, p = 0.56; NN: mean SI = 0.65 ± 0.12 at a threshold of 0.8, p = 0.41; AB: mean SI = 0.53 ± 0.20 at a threshold of 0.4, p = 0.10).

- On the NUHS Singapore cohort, SI values obtained from KNN are not significantly different from those obtained from the RF classifier (mean SI = 0.65 ± 0.26 at a threshold of 0.9, p = 0.15) but they are significantly higher than SI values obtained using NN (mean SI = 0.62 ± 0.27 at a threshold of 0.8, p = 0.009) and AB (mean SI = 0.55 ± 0.26 at a threshold of 0.4, p = 0.005).
- Similarly, on the UMC Utrecht cohort, SI values obtained from KNN are not significantly different from those obtained from the RF classifier (mean SI = 0.60 ± 0.23 at a threshold of 0.9, p = 0.5), but are significantly higher than SI values obtained using NN (mean SI = 0.59 ± 0.23 at a threshold of 0.7, p = 0.01) and AB (mean SI = 0.54 ± 0.28 at a threshold of 0.4, p = 0.02).

Therefore, while SI values obtained using KNN are always significantly higher than those of AB classifier, they are not significantly different from those of RF and NN classifiers for most of the cohorts.

LOCATE: additional experiments

Performance of LOCATE with simple linear iterative clustering (SLIC)

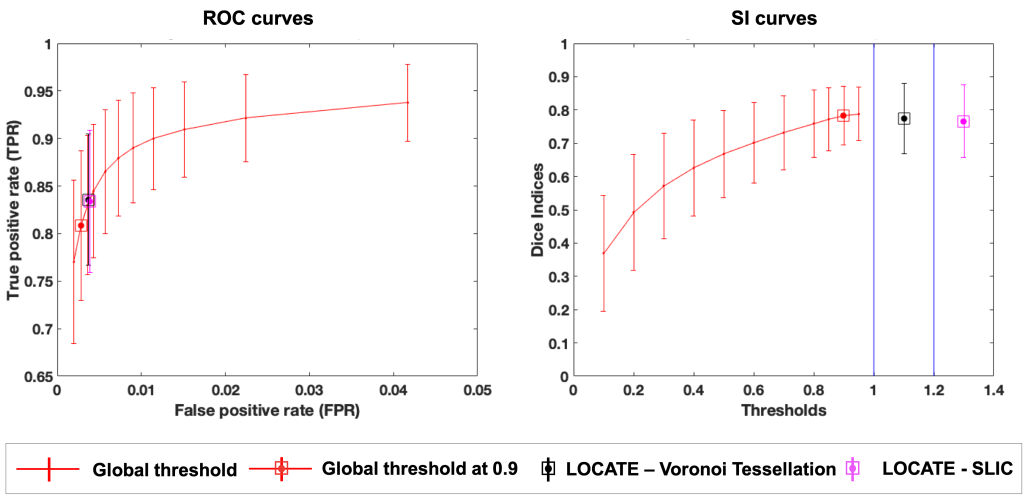

Figure S5: Comparison of ROC curves (left) and SI plots (right) for global thresholding (red) with LOCATE using Voronoi tessellation (black) and SLIC (magenta) on NDGEN dataset.

In order to test possible differences in LOCATE performance with respect to the tessellation method, we tested LOCATE using SLIC for forming sub-regions. For this experiment, we set the number of superpixels to 500, since this was comparable with the number of regions formed with Voronoi tessellation. Figure S5 shows ROC curves and SI curves for BIANCA using various global thresholds and LOCATE

using Voronoi tessellation and SLIC on NDGEN dataset. LOCATE gives similar true positive rates and SI values for both the tessellation methods, with negligible difference in the performance. This shows that LOCATE is robust with respect to the method used for forming sub-regions.

The paired t-test results show that the SI values obtained with LOCATE using Voronoi tessellation were significantly higher than those obtained using SLIC (LOCATE Voronoi tessellation SI = 0.77 ± 0.10, LOCATE SLIC SI = 0.76 ± 0.11, p = 0.003).

Figure S6 illustrates examples of LOCATE outputs using SLIC and Voronoi tessellation compared against the manual segmentation.

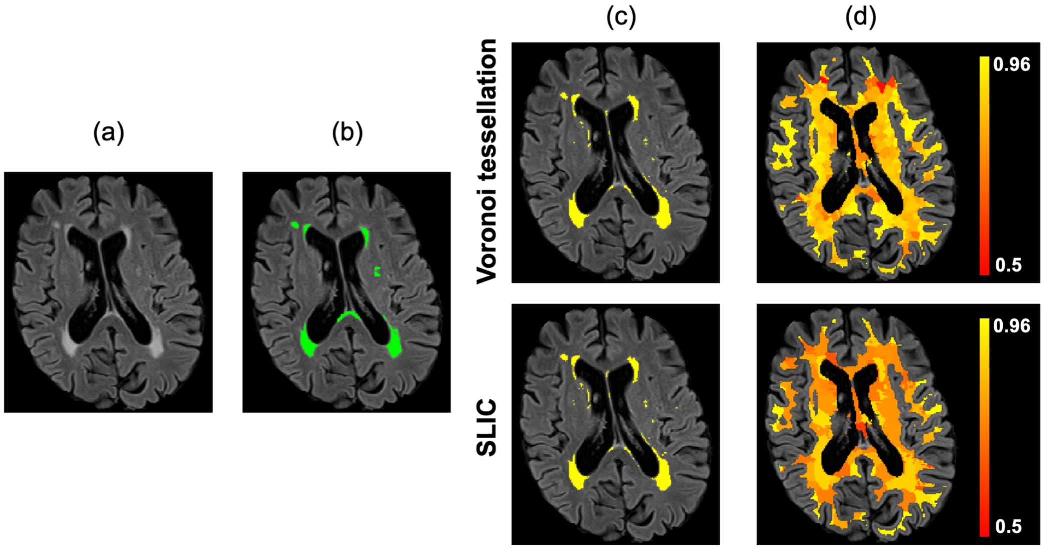

Figure S6: Results of LOCATE using Voronoi tessellation and SLIC for forming sub-regions. FLAIR (a) and manual segmentation (b) shown with LOCATE results (c) and thresholds map (d) using Voronoi tessellation (top row) and SLIC (bottom row).

Comparison of LOCATE performance in deep and periventricular regions

We used performance metrics such as TPR, FPR, SI and cluster-wise TPR for evaluating the performance of LOCATE. In order to determine the performance of LOCATE in deep and periventricular regions individually and to compare them, we evaluated the above metrics separately in deep and periventricular regions. We have already shown the cluster-wise TPR plots in the deep and periventricular regions for all datasets: NDGEN, OXVASC and MWSC (VU Amsterdam, NUHS Singapore, UMC Utrecht) in Figure 11 in main manuscript. Also, in Table 3 we have reported the main and interaction effects on cluster-wise TPR values by performing 2-way

repeated measures ANOVA. In this section, we provide similar plots and statistical results on the other measures: TPR, FPR and SI.

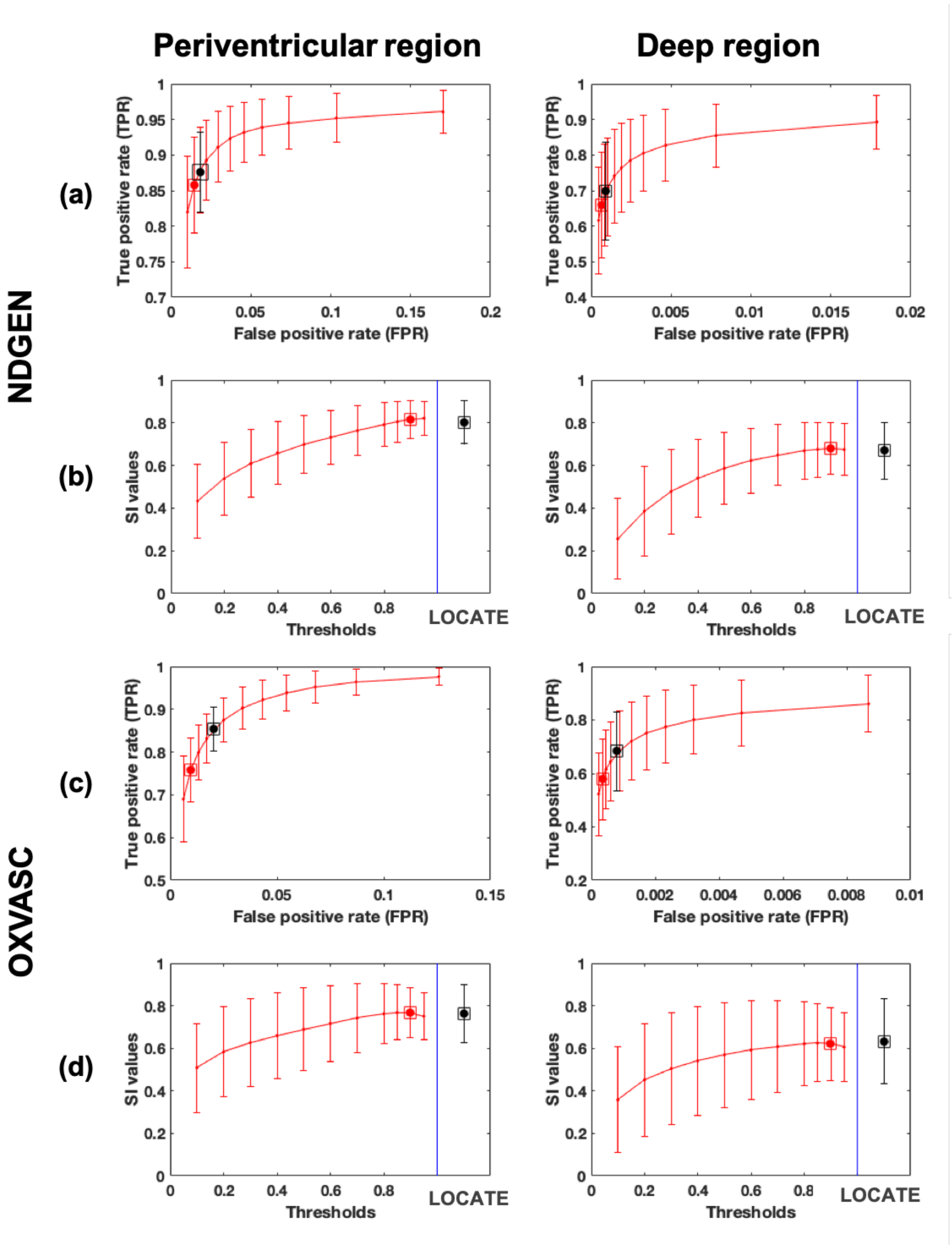

Figure S7: ROC curves (a, c) and SI curves (b, d) in periventricular (left) and deep (right) regions for LOCATE (black) and global thresholding (red) on NDGEN and OXVASC datasets.

Figure S7 shows the ROC curves and SI plots for NDGEN, OXVASC datasets. Similarly, figures S8 and S9 show the ROC and SI plots for 3 cohorts of MWSC dataset.

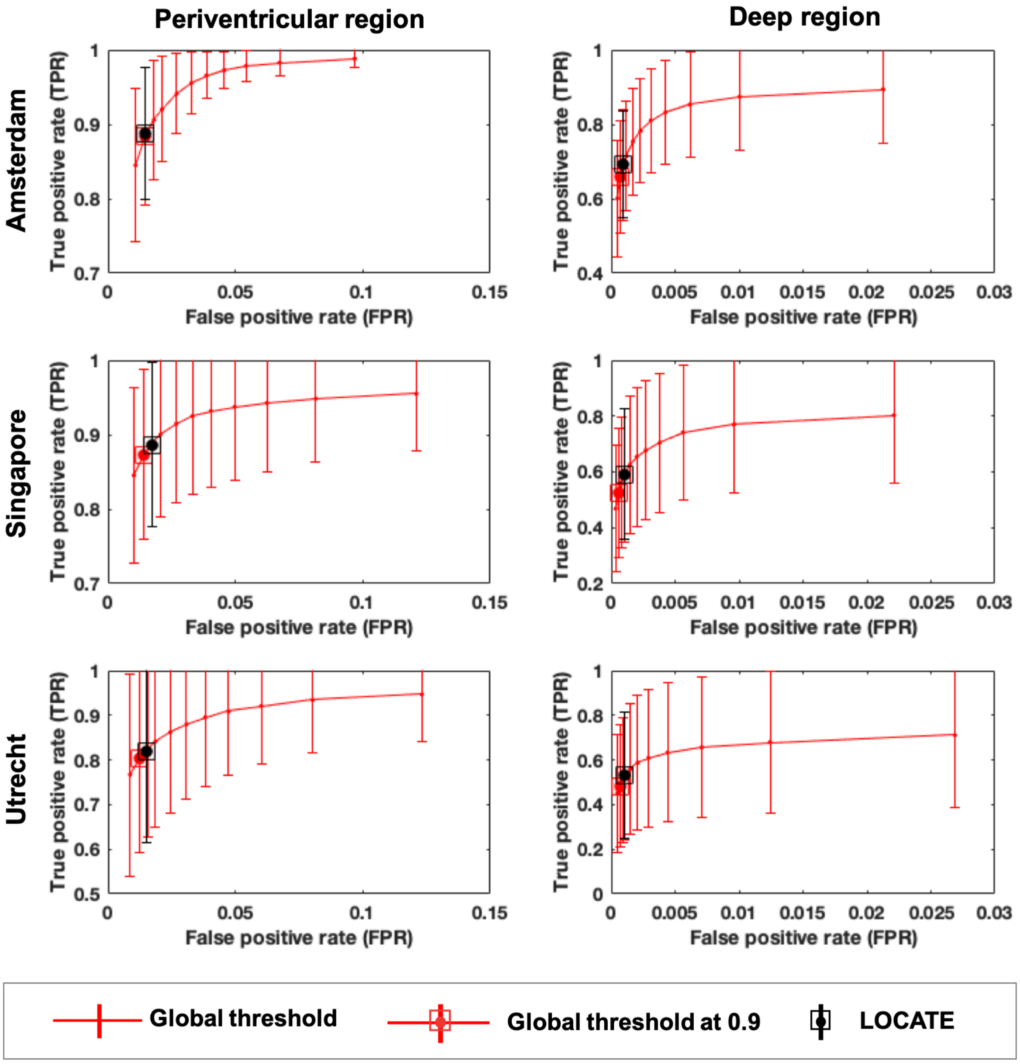

Figure S8: ROC curves in periventricular (left) and deep (right) regions for LOCATE (black) and global thresholding (red) on VU Amsterdam (top row), NUHS Singapore (middle row) and UMC Utrecht (bottom row) datasets.

We also performed 2-way repeated measures ANOVA considering region (deep and periventricular) and method (LOCATE and Global thresholding at 0.9) as independent factors and TPR, FPR and SI as the dependent measure. We determined the main effect of region and method, along with the effect of their interaction, on

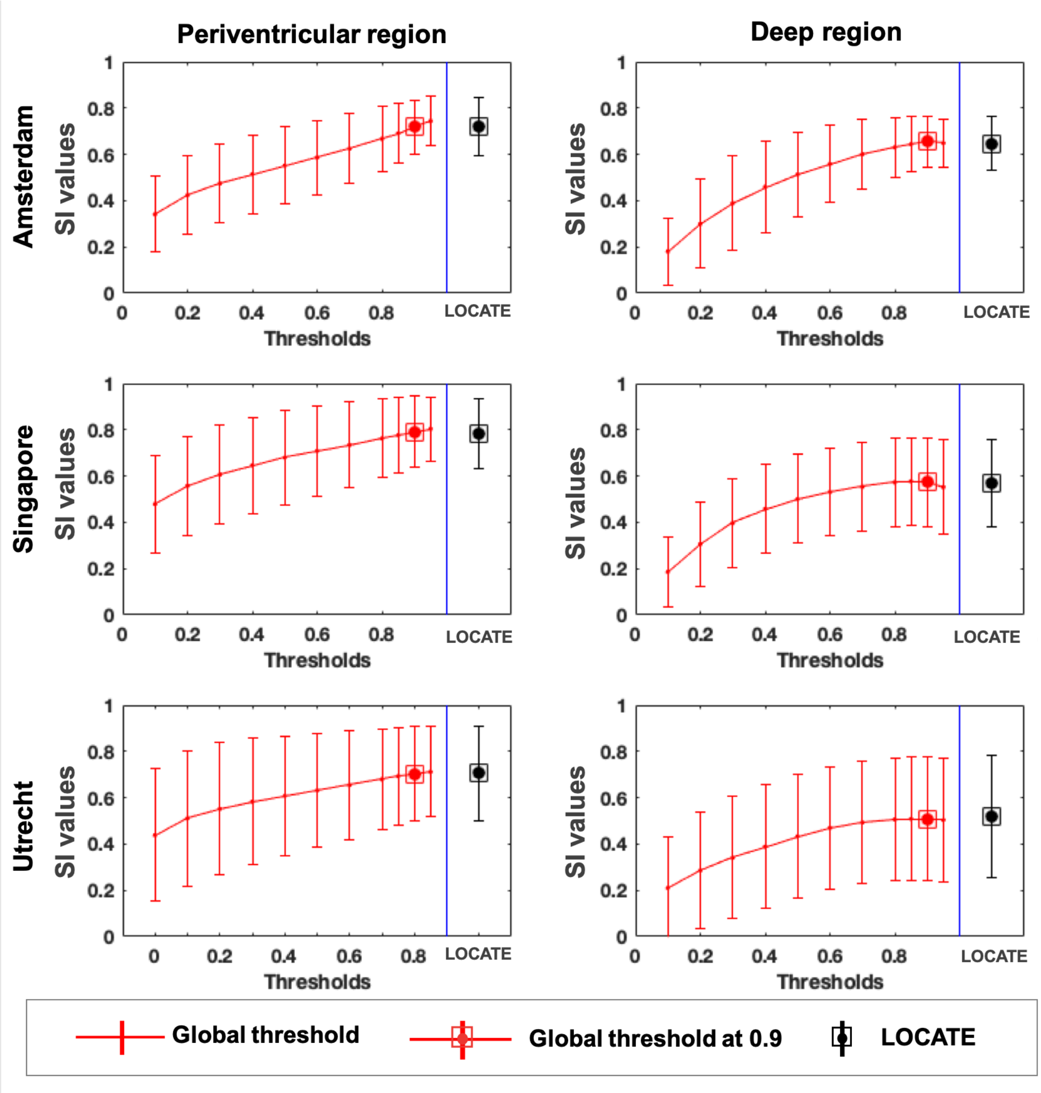

Figure S9: SI curves in periventricular (left) and deep (right) regions for LOCATE (black) and global thresholding (red) on VU Amsterdam (top row), NUHS Singapore (middle row) and UMC Utrecht (bottom row) datasets.

the individual measures. Tables S1, S2 and S3 report the descriptive statistics and ANOVA results for comparison of TPR, FPR and SI values respectively.

For TPR values, LOCATE provides significantly higher TPR values when compared with the global thresholding and TPR values are significantly higher in periventricular regions when compared to deep regions. In almost all datasets, the interaction effect is also significant indicating that the increase in TPR using LOCATE with respect to global threshold was significantly higher in deep regions compared to periventricular. Even though FPR values are negligible in general, they are higher

for LOCATE when compared to global thresholding and are higher in the periventricular regions for NDGEN, NUHS Singapore and UMC Utrecht datasets. We observed that the interaction effect is also significant in most of MWSC cohorts and NDGEN datasets. The main effect of method is not significant on SI values except for the NDGEN dataset. However, SI values are significantly higher in periventricular regions when compared with the deep regions for all the datasets.

Overall, we observed significant main effects of both method and region on the detection of true positive lesions. Additionally, in most of the datasets we observed a significant interaction effect. This shows that the improvement in detection of true positive lesions using LOCATE with respect to global thresholding is significantly higher in deep region compared to periventricular region.

			NDGEN	OXVASC	VU Amsterdam	NUHS Singapore	UMC Utrecht
Descriptive statistics	Deep region	LOCATE	0.70 ± 0.14	0.68 ± 0.15	0.69 ± 0.14	0.59 ± 0.23	0.53 ± 0.28
		Global 0.9	0.66 ± 0.15	0.57 ± 0.15	0.65 ± 0.15	0.52 ± 0.23	0.48 ± 0.27
	Peri-venticular region	LOCATE	0.88 ± 0.06	0.85 ± 0.05	0.89 ± 0.08	0.89 ± 0.11	0.82 ± 0.20
		Global 0.9	0.86 ± 0.07	0.76 ± 0.07	0.88 ± 0.09	0.87 ± 0.11	0.80 ± 0.21
ANOVA	Main effect of region (deep/PV regions)		**F(1,19)=43.1 p<0.001 η_p^2=0.694**	**F(1,19)=41.4 p<0.001 η_p^2=0.685**	**F(1,16)=19.3 p<0.001 η_p^2=0.547**	**F(1,19)=50.8 p<0.001 η_p^2=0.728**	**F(1,19)=46.7 p<0.001 η_p^2=0.711**
	Main effect of method (LOCATE/Global 0.9)		**F(1,19)=15.3 p=0.001 η_p^2=0.446**	**F(1,19)=61.1 p<0.001 η_p^2=0.763**	**F(1,16)=116.7 p<0.001 η_p^2=0.879**	**F(1,19)=54.1 p<0.001 η_p^2=0.740**	**F(1,19)=23.6 p<0.001 η_p^2=0.554**
	Interaction (region * method)		**F(1,19)=49.2 p<0.001 η_p^2=0.663**	**F(1,19)=49.3 p<0.001 η_p^2=0.721**	F(1,16)=0.51 p=0.48 η_p^2=0.031	**F(1,19)=27.8 p<0.001 η_p^2=0.594**	**F(1,19)=12.2 p=0.002 η_p^2=0.391**

Table S1: Descriptive statistics and ANOVA results for comparison of TPR values for global threshold of 0.9 and LOCATE in periventricular and deep regions for MWSC datasets.

			NDGEN	OXVASC	VU Amsterdam	NUHS Singapore	UMC Utrecht
Descriptive statistics	Deep region	LOCATE	10.00×10^{-4}	11.3×10^{-4}	9.07×10^{-4}	10.00×10^{-4}	11.00×10^{-4}
		Global 0.9	7.00×10^{-4}	8.40×10^{-4}	7.00×10^{-4}	6.00×10^{-4}	7.00×10^{-4}
	Peri-venticular region	LOCATE	1.9×10^{-2}	2.0×10^{-2}	1.5×10^{-2}	1.7×10^{-2}	1.5×10^{-2}
		Global 0.9	1.5×10^{-2}	0.9×10^{-2}	1.5×10^{-2}	1.4×10^{-2}	0.13×10^{-2}
ANOVA	Main effect of region (deep/PV regions)		**F(1,18)=42.5**<br>**p<0.001**<br>η_p^2**=0.703**	F(1,16)=0.55<br>p=0.47<br>η_p^2=0.033	**F(1,18)=24.3**<br>**p<0.001**<br>η_p^2**=0.574**	**F(1,16)=113.2**<br>**p<0.001**<br>η_p^2**=0.876**	**F(1,16)=34.3**<br>**p<0.001**<br>η_p^2**=0.682**
	Main effect of method (LOCATE/Global 0.9)		**F(1,18)=28.9**<br>**p=0.001**<br>η_p^2**=0.616**	F(1,16)=1.12<br>p=0.3<br>η_p^2=0.065	F(1,18)=0.07<br>p=0.8<br>η_p^2=0.004	**F(1,16)=34.6**<br>**p<0.001**<br>η_p^2**=0.684**	**F(1,16)=12.6**<br>**p=0.003**<br>η_p^2**=0.440**
	Interaction (region * method)		**F(1,18)=23.9**<br>**p<0.001**<br>η_p^2**=0.570**	F(1,16)=0.34<br>p=0.57<br>η_p^2=0.021	F(1,18)=2.39<br>p=0.139<br>η_p^2=0.118	**F(1,16)=23.2**<br>**p=0.001**<br>η_p^2**=0.592**	**F(1,16)=12.1**<br>**p=0.003**<br>η_p^2**=0.430**

Table S2: Descriptive statistics and ANOVA results for comparison of FPR values for global threshold of 0.9 and LOCATE in periventricular and deep regions for MWSC datasets.

			NDGEN	OXVASC	VU Amsterdam	NUHS Singapore	UMC Utrecht
Descriptive statistics	Deep region	LOCATE	0.67 ± 0.14	0.63 ± 0.20	0.65 ± 0.11	0.57 ± 0.19	0.52 ± 0.26
		Global 0.9	0.68 ± 0.13	0.62 ± 0.18	0.65 ± 0.11	0.57 ± 0.20	0.50 ± 0.27
	Peri-venticular region	LOCATE	0.80 ± 0.10	0.76 ± 0.14	0.72 ± 0.12	0.79 ± 0.14	0.70 ± 0.20
		Global 0.9	0.81 ± 0.09	0.76 ± 0.12	0.72 ± 0.12	0.79 ± 0.15	0.70 ± 0.20
ANOVA	Main effect of region (deep/PV regions)		**F(1,19)=57.9**<br>**p<0.001**<br>η_p^2**=0.753**	**F(1,19)=22.4**<br>**p<0.001**<br>η_p^2**=0.583**	**F(1,16)=4.45**<br>**p=0.048**<br>η_p^2**=0.190**	**F(1,19)=28.9**<br>**p<0.001**<br>η_p^2**=0.604**	**F(1,19)=30.1**<br>**p<0.001**<br>η_p^2**=0.613**
	Main effect of method (LOCATE/Global 0.9)		**F(1,19)=5.4**<br>**p=0.031**<br>η_p^2**=0.222**	F(1,19)=0.09<br>p=0.76<br>η_p^2=0.006	F(1,16)=0.82<br>p=0.38<br>η_p^2=0.041	F(1,19)=0.72<br>p=0.41<br>η_p^2=0.037	F(1,19)=0.76<br>p=0.4<br>η_p^2=0.039
	Interaction (region * method)		F(1,19)=0.24<br>p=0.63<br>η_p^2=0.013	F(1,19)=2.52<br>p=0.13<br>η_p^2=0.136	F(1,16)=1.14<br>p=0.3<br>η_p^2=0.056	F(1,19)=0.32<br>p=0.58<br>η_p^2=0.016	F(1,19)=2.66<br>p=0.12<br>η_p^2=0.123

Table S3: Descriptive statistics and ANOVA results for comparison of SI values for global threshold of 0.9 and LOCATE in periventricular and deep regions for MWSC datasets.
